# Molecular Network Guided Cataloging of the Secondary Metabolome of Selected Egyptian Red Sea Soft Corals

**DOI:** 10.3390/md20100630

**Published:** 2022-10-01

**Authors:** Nesrine M. Hegazi, Tarik A. Mohamed, Hamada H. Saad, Montaser A. Al-Hammady, Taha A. Hussien, Mohamed-Elamir F. Hegazy, Harald Gross

**Affiliations:** 1 Department of Phytochemistry and Plant Systematics, National Research Centre, 33 El-Bohouth St., Dokki, Giza 12622, Egypt; 2 Chemistry of Medicinal Plants Department, National Research Centre, 33 El-Bohouth St., Dokki, Giza 12622, Egypt; 3 Department of Pharmaceutical Biology, Pharmaceutical Institute, University of Tübingen, Auf der Morgenstelle 8, 72076 Tübingen, Germany; 4 National Institute of Oceanography and Fisheries (NIOF), Cairo 11516, Egypt; 5 Pharmacognosy Department, Faculty of Pharmacy, Sphinx University, New Assiut City 71515, Egypt

**Keywords:** Egyptian soft corals, metabolome profiling, molecular networking

## Abstract

Soft corals are recognized as an abundant source of diverse secondary metabolites with unique chemical features and physiologic capabilities. However, the discovery of these metabolites is usually hindered by the traditional protocol which requires a large quantity of living tissue for isolation and spectroscopic investigations. In order to overcome this problem, untargeted metabolomics protocols have been developed. The latter have been applied here to study the chemodiversity of common Egyptian soft coral species, using only minute amounts of coral biomass. Spectral similarity networks, based on high-resolution tandem mass spectrometry data, were employed to explore and highlight the metabolic biodiversity of nine Egyptian soft coral species. Species-specific metabolites were highlighted for future prioritization of soft coral species for MS-guided chemical investigation. Overall, 79 metabolites were tentatively assigned, encompassing diterpenes, sesquiterpenes, and sterols. Simultaneously, the methodology assisted in shedding light on newly-overlooked chemical diversity with potential undescribed scaffolds. For instance, glycosylated fatty acids, nitrogenated aromatic compounds, and polyketides were proposed in *Sinularia leptoclados*, while alkaloidal terpenes and *N*-acyl amino acids were proposed in both *Sarcophyton roseum* and *Sarcophyton acutum.*

## 1. Introduction

Egypt, with its exclusive geographical location spanning over 3000 km of coastal area, offers numerous distinguishable habitats with abundant biodiversity. The Red Sea is one of the most biodiverse areas, harboring multitudes of corals and sponges [1]. Among coral species worldwide, 40% are inhabitants of the Red Sea [2]. 

Soft corals represent an outstanding natural resource harboring diverse classes of natural products with a broad spectrum of biological properties [2]. In addition, numerous extracted chemotypes from soft corals have been shown to be primarily involved in chemical defense mechanisms of their producers, as well as their adaptation strategy [3]. 

Generally, soft corals are known to produce a wide variety of terpenes [4]. Among these, diterpenes are the most prominent metabolites featuring different skeletons such as cembranes, norcembranes, xeniaenes, briaranes, and eunicellins. Cembrane diterpenes, the most explored molecular family, are produced by *Sinularia* and *Lobophytum*, and are enriched in *Sarcophyton* species [5,6,7]. As a result of the promising pharmacological profiles presented by such scaffolds, the possible harnessing of cembrane diterpenes as potential antitumor agents represents significant potential [8,9]. Additionally, eunicellin-based diterpenes have also displayed ecological, agrochemical, and pharmacological significance [10]. This type of diterpenoids is the representative class of the genus *Cladiella* [11]. Alternatively, soft corals of the genera *Sinularia*, *Nephthea*, *Lemenalia*, and others, are producers of sesquiterpenes and norsesquiterpenes, with highly rearranged skeletons [12]. 

In addition to the predominant terpenes produced by the corals, they have been recognized to produce other natural product chemotypes. Recently, ribosomal peptides were isolated and identified from scleractinian corals [13], along with acylated glycolipids [14], and alkaloids [15]. However, as the recent genetic investigations of corals suggest [3,16], to date, the reported chemistry merely represents only a fraction of the metabolites that are yet to be discovered. 

Recently, advances in metabolomics tools have enabled the development of a comprehensive chart of the metabolic chemical space of living organisms [17]. The exploration of hundreds of metabolites is now feasible with the recent advances in the metabolomic platforms along with the opportunity to compare specimens simultaneously in an untargeted manner, allowing for the recognition of variability and similarity among samples [18].

Recently, untargeted metabolomic approaches in combination with multivariate data analysis were successfully applied to study cembrane diterpenes of different *Sarcophyton* species growing under different environmental and chemical conditions [2,19,20]. During this study, the superiority of the LC-MS in the discriminative coverage of coral species based on their chemical profiles, became apparent [2]. Subsequently, feature-based molecular networking was employed using the GNPS platform (the Global Natural Products Social Molecular Networking) for the identification of distinct chemical features among different clades of *Sarcophyton glaucum* [21]. 

Analogously, our primary aim was to explore and highlight the metabolic biodiversity offered by common Egyptian soft corals with the aid of untargeted metabolomics. Furthermore, species-specific metabolites have been highlighted for the future prioritization of soft coral species for MS-guided chemical investigation. Specimens studied included three *Sinularia* species (*Si. brassica* (formerly known as *Si. dura*)*, Si. leptoclados,* and *Si. gardineri*), three *Sarcophyton* species (*Sa. roseum* (formerly known as *Sa. convolutum*), *Sa. ehrenbergi*, and *Sa. acutum*), along with *Cladiella pachyclados*, *Litophyton mollis* (formerly known as *Nephthea mollis*) and *Lobophytum pauciflorum.* Molecular networking was consulted to visually dissect the chemical similarities and differences among the specimens through the GNPS platform based on tandem mass spectrometric data (HRMS/MS).

## 2. Results and Discussion

Since the ultimate objective of this study was to broadly catalog the secondary metabolites of some common Egyptian soft corals with an emphasis on unearthing new chemistries, metabolomic-based indexing was thought to be the best means to interrogate the metabolomes. In concert, several dereplication algorithms have lately emerged to partially address the challenging annotation step in data analysis which contributes to the understanding of the complex metabolomics data sets, potentially revealing previously undiscovered metabolites [22]. In this regard, UPLC-HRMS/MS analysis in conjunction with feature-based molecular networking (FBMN) was harnessed to describe the nature and extent of the chemical diversity of the secondary metabolomes of such a defined set of soft corals. 

**Figure 1:**
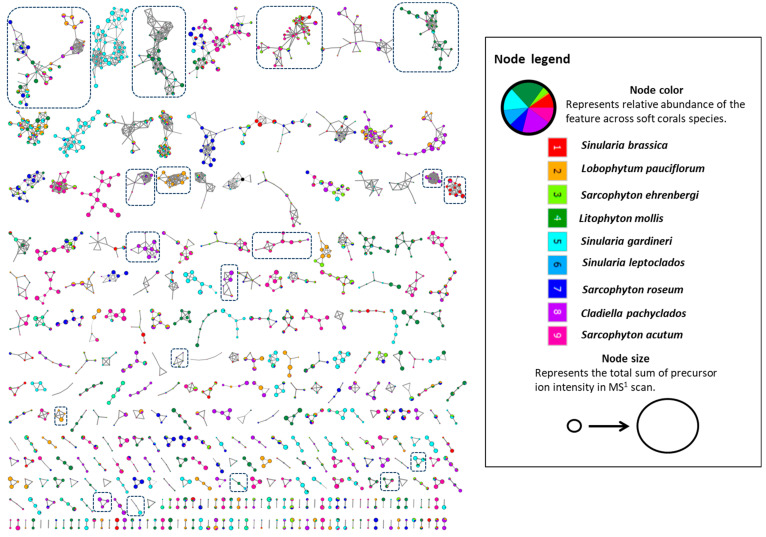
The feature-based molecular network created using MS/MS data in the positive ionization mode from the soft coral extracts. The network displays nodes as a pie chart to reflect the relative abundance of each ion in each of the extracts.

UPLC-HRMS/MS analysis in the positive ionization mode of the selected Egyptian soft corals revealed distinct chemical profiles as seen in their respective base peak chromatograms (Supplementary Figure S1). The generation of an FBMN using the positive profiles portrayed a global overview of the detected chemical space and the metabolite distribution in the soft corals under investigation [23]. Networks were found that describe 3442 features (represented as nodes), with 1904 connected features in 480 clusters and the rest as singletons (Figure 1). Annotated clusters within the network are shown in boxes (Figure 1) which will be described in detail in terms of class identities in the upcoming sections. 

Metabolite dereplication was principally based on their observed retention times, chemical formulas, and fragmentation behavior. Furthermore, the metabolite identification process was augmented with the FBMN and *in silico* fragmentation trees proposed by Sirius [24], in conjunction with the MarinLit database (https://marinlit.rsc.org/, accessed on 1 August 2022).

**Figure 2:**
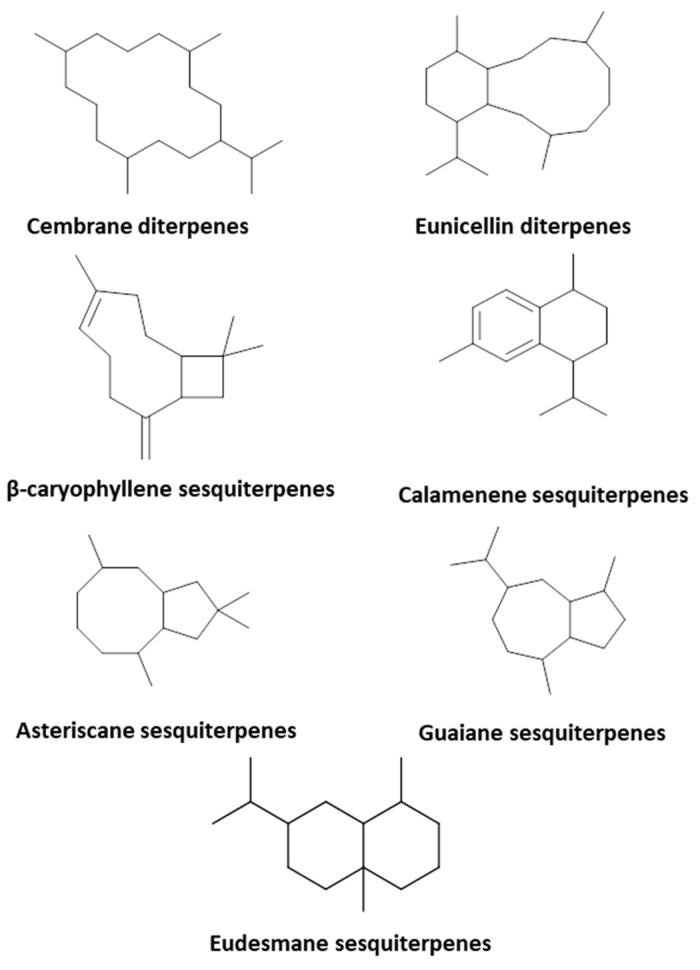
Diterpene and sesquiterpene skeletons identified in the studied soft coral species.

In-depth exploration of the differences between the studied species disclosed the existence of cembrane diterpenes in almost all species. In line with previous reports [11,25], eunicellin diterpenes occurred exclusively in *Cladiella pachyclados* but are reported here for the first time in *Lobophytum pauciflorum* (Figure 4). Similarly, sesquiterpenes with rearranged skeletons existed predominantly in *Litophyton mollis* and were less frequently encountered in *Sinularia* species (Figure 2).

Overall, 79 metabolites were tentatively assigned, mainly belonging to the terpene class encompassing diterpenes (cembrane and eunicellin types), sesquiterpenes (β-caryophyllene, asteriscane, eudesmane, guaiazulene, and calamenene types), and sterols. Detailed LC and MS information of the annotated metabolites is tabulated in Table S1. However, it is worthy to mention that the relative and/or absolute configuration of the annotated entities cannot be determined unless other appropriate spectroscopic techniques are implemented.

### 2.1. Cembrane Diterpenes

**Figure 3:**
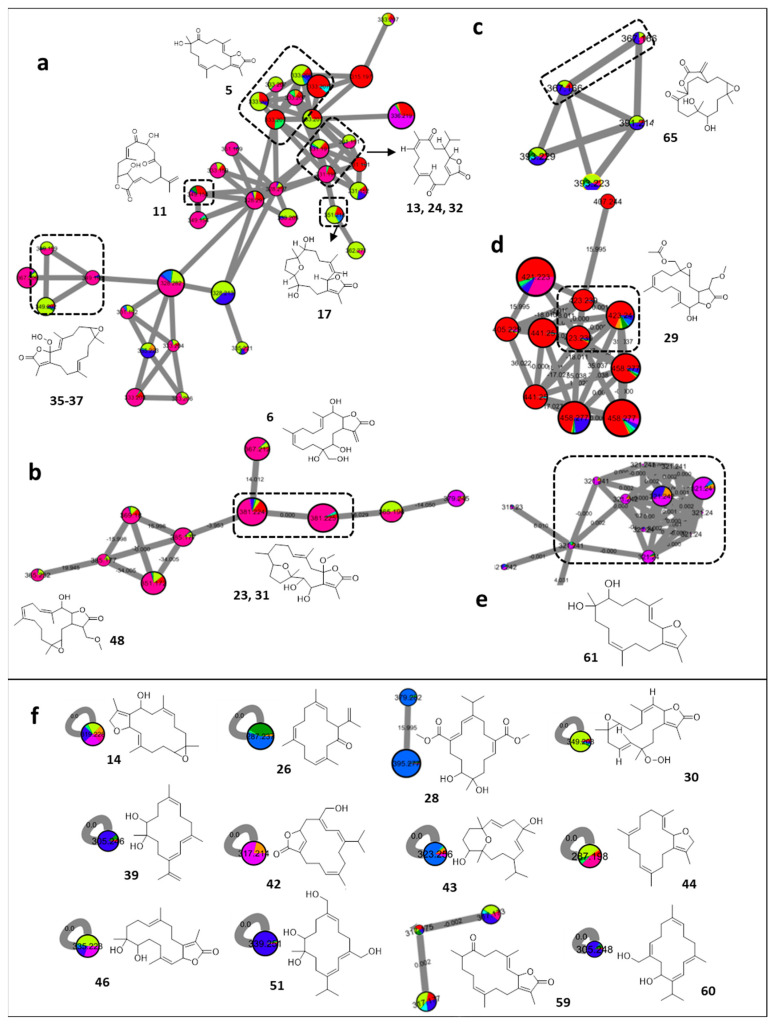
Annotated cembrane diterpenes and their distribution in the FBMN. Node color corresponds to color codes described in Figure 1.

Detailed analysis of the FBMN uncovered the richness of the soft coral species with cembrane diterpenes (Figure 3). Such entities have previously been obtained from several soft coral genera, including *Sarcophyton*, *Sinularia*, *Lobophytum*, *Eunicea*, *Clavularia*, and from other octocorals [26]. Ecologically, cembranoids are thought to act as defensive compounds protecting soft corals from predators, bacteria, and other organisms [26]. In addition, several in vitro studies have documented their broad biological efficacy in anti-inflammatory, anticancer, antibacterial, antiviral, neuroprotective, and cytotoxic assays [26,27]. Interestingly, it was recently discovered that dolphins use these metabolites for self-medication against skin infections by rubbing their skin against specific soft coral species (i.e., *Sarcophyton* sp.). Tracing this behavior, cembranoid diterpenes (sarcophine/sarcophytolide/sarcophytolide B or C) could be linked as underlying metabolites, since they protect the dolphins from dermal pathogens [28].

Manual annotation, supported with GNPS cross-referencing, facilitated the deconvolution of several clusters as cembrane diterpenes and expectedly revealed isomeric structural diversity (Figure 3). Their MS^2^ spectra showed the typical fragmentation pattern of terpenes with fragments separated by 12–14 Da, besides the common neutral losses of H_2_O (−18 Da) and CO (−28 Da), depending on the skeleton of the diterpene. In addition, the loss of 71 Da, representing the expulsion of an acyloxy group (C_3_H_5_O_2_), was regarded as a further informative fragment highlighting the γ-lactone ring-derived architectures. While cembranes with a hydroperoxy group showed the successive losses of –OH (17 Da) and –O (16 Da), those decorated with an acetyl group exhibited the typical loss of 42 Da [2]. Although the fragmentation behavior of the cembrane diterpenes was quite similar and showed the same fragments, there were subtle differences in their abundance attributed to their clustering into different groups, or even as single nodes in the MN, with a cosine score of 0.7 [21]. 

The major cluster of cembrane diterpenes demonstrated their distribution across *Sinularia brassica*, *Sarcophyton ehrenbergi*, and *Sarcophyton acutum* (Figure 3a). The cluster encompassed several isomers of a previously characterized hexahydrohydroxytetramethylcyclotetradecafurandione (**5**, *m/z* 333.2067 [M + H]^+^, C_20_H_28_O_4_) from *Sarcophyton ehrenbergi* [29]. Three connected features were hypothesized to depict the isomeric features of sarcostolide D [30] (**13**, **24**, and **32**, *m/z* 331.1897 [M + H]^+^, C_20_H_26_O_4_) and another feature was hypothesized to be sarcoehrenbergilid C [7] (**17**, *m/z* 351.2160 [M + H]^+^, C_20_H_30_O_5_). Within the same cluster, gyrosanolide A (**11**, *m/z* 349.2002 [M + H]^+^, C_20_H_26_O_4_) was proposed as a uniquely morphed cembrane-derived scaffold formerly described in *Sinularia gyrosa* [31]. In addition, isomers of hydroperoxysarcophine (**35** and **37**, *m/z* 349.1998 [M + H]^+^, C_20_H_28_O_5_), previously reported to occur in the soft corals *Sarcophyton infundibuliforma* [32] and *Lobophytum crassum* [33], respectively, were also proposed to be interlinked to the same ion cluster. 

Similarly, an additional group of cembrane diterpenes sharing the same distribution pattern among the species (Figure 3b) were uncovered, and were proposed to consist of sinularolide A [34] (**6**, *m/z* 367.2111 [M + H]^+^, C_20_H_30_O_6_), isomers of briaviodiol A [35] (**23** and **31**, *m/z* 381.2259 [M + H]^+^, C_21_H_32_O_6_) and durumolide Q [36] (**48**, *m/z* 365.2316 [M + H]^+^, C_21_H_32_O_5_).

Furthermore, sinulaparvalide A [37] (**65**, *m/z* 367.211 [M + H]^+^, C_20_H_30_O_6_) was proposed as a metabolite of *Sarcophyton roseum*, and found to be associated with a further set of descendant ions possessing mass differences of +24 and +26 Da. The latter tentative derivatives of **65,** which are larger in size, have not been previously described and represent possibly new congeners (Figure 3c).

The *Sinularia brassica* profile was proposed to harbor an exclusive group of structurally related cembrane diterpenes (Figure 3d), and among them, durumolide O [36] (**29**, *m/z* 423.2375 [M + H]^+^, C_21_H_32_O_5_), was annotated. The associated features were proposed to identify possibly new congeners with variable structural modifications such as dehydration, hydration, hydroxylation, or hydroperoxylation, as implied from the edge connections of −18 Da, +18 Da, +16 Da, or +17 Da, respectively. 

Similarly, *Cladiella pachyclados* appeared to produce numerous isomers of dihydroxydeepoxysarcophytoxide [38] (**61**, *m/z* 321.2419 [M + H]^+^, C_20_H_32_O_3_) (Figure 3e).

Lastly, a number of cembrane diterpenes were extracted as either a cluster of two nodes or as singletons as shown in Figure 3f and Table S1. 

### 2.2. Eunicellin Diterpenes

Alongside the abundant cembrane diterpenes, numerous eunicellin-based scaffolds were annotated exclusively in *Cladiella pachyclados* and *Lobophytum pauciflorum*. In line with former investigations [11], eunicellins are recognized as the prevailing terpenes in *Cladiella*. Eunicellin-derived diterpenes are always found in soft corals as a principal source, in contrast to others such as plants and microbes. Corals, particularly *Cladiella*, *Eunicella*, *Briareum*, and *Muricella*, are regarded as mega-producing genera of diverse eunicellin diterpenoids [39].

The architecture of eunicellin diterpenes is fundamentally constructed on the basis of a cladiellane diterpene frame and appended with a C-2, C-9 ether bridge to install a tetrahydrofuran ring. Structurally, they are believed to be the descendants of the cembrane diterpenes framed via a 2, 11-cyclization event [39]. As a result of the unusual structural features they can be decorated with, and, in turn, the compelling pharmacological activities, eunicellin diterpenes have captured the interest of both chemists and biologists in diverse chemical biology endeavors [39,40].

**Figure 4:**
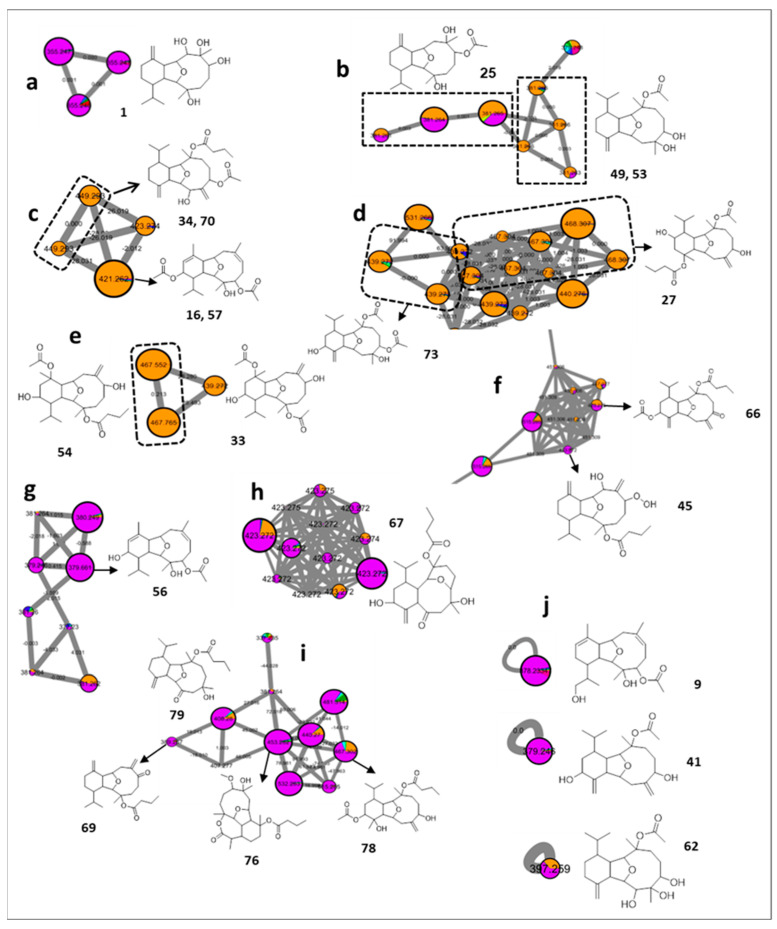
Annotated eunicellin diterpenes and their distribution in the FBMN. Node color corresponds to color codes described in Figure 1.

The FBMN revealed the presence of multiple clusters coding for the eunicellin terpenes (Figure 4). Their scattered appearance in several clusters was attributed to the relative differences in the abundance of the fragments (Figure S2), as previously mentioned [21].

The first assigned putative eunicellin diterpene was sclerophytin D [41] (**1**, *m/z* 355.2484 [M + H]^+^, C_20_H_34_O_5_) identified in *Cladiella pachyclados* (Figure 4a). A further pair of eunicellin-related features were proposed from a cluster mainly derived from *Cladiella pachyclados* and *Lobophytum pauciflorum* (Figure 4b). The tentative identification of such ions led to the proposition of the presence of sclerophytin B [42] (**25**, *m/z* 381.2627 [M + H]^+^, C_22_H_36_O_5_) with two additional isomeric forms including the structurally related sclerophytin E [43] (**49** and **53**, *m/z* 381.2633 [M + H]^+^, C_22_H_36_O_5_) (Figure 4b). 

Interestingly, the MN unequivocally delineated *Lobophytum pauciflorum* as a prolific source of eunicellin diterpenes (Figure 4c–e). Cluster **4c** was proposed to include astrogorgin B [44] (**16** and **57**, *m/z* 421.2581 [M + H]^+^, C_24_H_36_O_6_), connected with an edge of 28 Da to litophynol A acetate isomers [45] (**34** and **70**, *m/z* 449.2890 [M + H]^+^, C_26_H_40_O_6_), as reflected through their comparative elemental composition (+C_2_H_4_). Similarly, cluster **4d** was proposed to demonstrate several isomeric forms of pachycladin B [11] (**27**, *m/z* 467.2999 [M + H]^+^, C_26_H_42_O_7_), and cladieunicellin N [46] (**73**, *m/z* 439.2684 [M + H]^+^, C_24_H_38_O_7_), while klysimplexin E [47] (**33**, *m/z* 439.2687 [M + H]^+^, C_24_H_38_O_7_), and klysimplexin C [47] (**54**, *m/z* 467.2995 [M + H]^+^, C_26_H_42_O_7_) constituted cluster **4e**. 

As expected, the prevalence of the eunicellin diterpene in *Cladiella pachyclados* was proposed to be exemplified by the known oxylitophynol [45] (**45**, *m/z* 423.2748 [M + H]^+^, C_24_H_38_O_6_), and its characterized peroxy derivative [48] (**66**, *m/z* 447.2743 [M + H]^+^, C_26_H_38_O_6_), which was originally reported from the soft coral *Litophyton viscudium* (Figure 4f). 

In a similar fashion, and foremost in *Cladiella pachyclados*, three additional clusters were successfully tracked down (Figure 4g,h,i). The first and second groups were proposed to uncover astrogorgin L [44] (**56**, *m/z* 379.2471 [M + H]^+^, C_20_H_35_O_5_), and numerous isomers of cladieunicellin K [49] (**67**, *m/z* 423.2742 [M + H]^+^, C_24_H_38_O_6_), respectively. The third putatively defined set of eunicellin-derived features included litophynin G [50] (**69**, *m/z* 389.2682 [M + H]^+^, C_24_H_36_O_4_), briarellin P [51] (**76**, *m/z* 453.2837 [M + H]^+^, C_25_H_40_O_7_), simplexin P [52] (**78**, *m/z* 467.2999 [M + H]^+^, C_26_H_42_O_7_), and australin G [53] (**79**, *m/z* 407.2782 [M + H]^+^, C_24_H_38_O_5_).

Application of the same strategy on the single nodes also led to the tentative assignment of multiple singletons belonging to *Cladiella pachyclados*, as hirsutalin G [54] (**9**, *m/z* 379.2476 [M + H]^+^, C_22_H_34_O_5_), klymollin S [55] (**41**, *m/z* 379.2474 [M + H]^+^, C_22_H_34_O_5_), and sclerophytin C [43] (**62**, *m/z* 397.2579 [M + H]^+^, C_22_H_36_O_6_) (Figure 4j).

### 2.3. Sesquiterpenes

**Figure 5:**
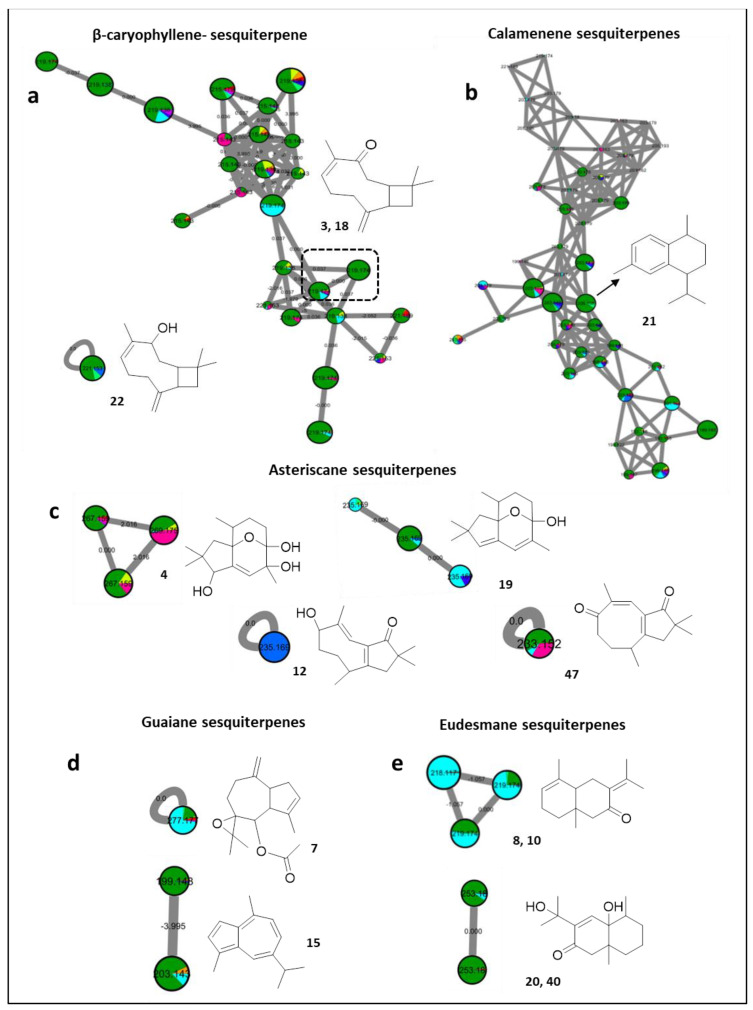
Annotated sesquiterpenes and their distribution in the FBMN. Node color corresponds to color codes described in Figure 1.

Different from the prevalent diterpenes, which were present in almost all the studied species, sesquiterpenes can only be readily predicted in *Litophyton mollis*, and sporadically in samples of the genus *Sinularia* (Figure 5).

Marine-derived sesquiterpenes embody a vital class of natural products framing countless scaffolds with a diverse array of bioactivity. The biological effects of marine sesquiterpenes were broadly detailed to span antitumor, antibacterial, antiviral, antifungal, immunosuppressive, cytotoxic, and insecticidal activities [56,57].

As illustrated from the FBMN, sesquiterpenes with various carbon skeletons were mostly proposed from the extract of *Litophyton mollis*. The first instance can be visualized by a group of features, namely, β-caryophyllene-based sesquiterpenes (Figure 5a). Such ions were proposed to include buddledin C/D [58] (**3** and **18**, *m/z* 219.1742 [M + H]^+^, C_15_H_23_O) in addition to numerous isomers (Figure 5a), while suberosol C [58] (**22**, *m/z* 221.1893 [M + H]^+^, C_15_H_24_O) was deciphered as a self-looped node, and was also found to exist in *Sinularia gardineri*, and *Sinularia leptoclados*.

Similarly, a large isomeric diversity was proposed for a calamenene-type sesquiterpene [59] (**21**, *m/z* 203.1789 [M + H]^+^, C_15_H_22_), previously characterized as (+)-*trans*-calamenene from *Nephthea erecta* (Figure 5b).

Furthermore, the MN suggests the additional occurrence of asteriscane sesquiterpenes in *Sarcophyton acutum*, *Lobophytum pauciflorum*, and *Sinularia leptoclados* (Figure 5c). This includes capillosanane J [60] (**4**, *m/z* 269.1748 [M + H]^+^, C_15_H_24_O_4_) grouped with an unknown pair of dehydrogenated derivatives, and isomers of capillosanane M [60] (**19**, *m/z* 235.1686 [M + H]^+^, C_15_H_22_O_2_), while, both capillosanane D [60] (**12**, *m/z* 235.1690 [M + H]^+^, C_15_H_20_O_2_), occurring exclusively in *Lobophytum pauciflorum*, and capillosanane F [60] (**47**, *m/z* 233.1528 [M + H]^+^, C_15_H_22_O_2_), appeared as scattered single nodes (Figure 5c).

Moreover, a further family of guaiane sesquiterpenes was proposed, consisting of guaiane spiroazulene **7** [61] (*m/z* 277.1775 [M + H]^+^, C_17_H_24_O_3_) and the aromatic cuteazul [62] (**15**, *m/z* 199.1478 [M + H]^+^, C_15_H_18_) (Figure 5d). In addition, the presence of eudesmane-based sesquiterpenes, such as isomers of naphthalenones **8** and **10** [63] (*m/z* 219.1744 [M + H]^+^, C_15_H_22_O), and oxoeudesmendiol [60,64] (**20** and **40**, *m/z* 253.1791 [M + H]^+^, C_15_H_24_O_3_) (Figure 5e), was suggested.

### 2.4. Sterols

Soft corals, in addition to being a prolific source of bioactive sesquiterpenes and diterpenes, are also known for their biosynthesis of polar polyhydroxy steroids. Typically, coral-derived sterols are structurally characterized by a 3β-hydroxy-Δ^5^- (or Δ^0^-) cholestane nucleus embedded with a C_8_−C_10_ side chain [65]. Coral-derived oxysterols are believed to be involved in the chemical defense against competitor reef organisms and predators. Therefore, interest is constantly growing to expand their physiological and pharmacological profiles. Moreover, they have demonstrated many biological properties such as cytotoxicity, lowering cholesterol biosynthesis, and inhibition of cancer [66].

**Figure 6:**
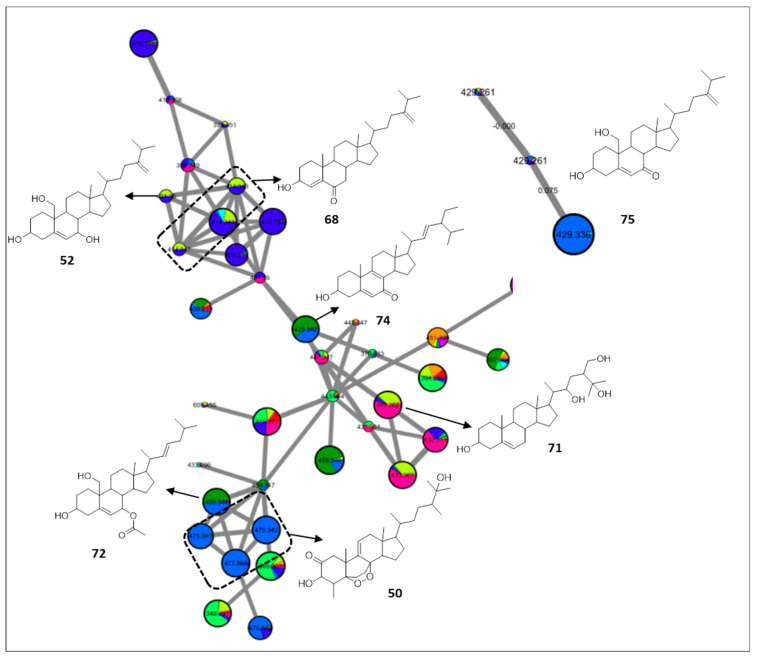
Annotated sterols and their distribution in the FBMN. Node color corresponds to color codes described in Figure 1.

The FBMN unearthed the presence of oxysterols in almost all specimens with a typical fragmentation pattern of successive loss of H_2_O and subsequent loss of the side chain. A major cluster of oxysterols was proposed with prevalence in *Lobophytum pauciflorum*, and consisted of an epidioxy ergosterol analogue [67] (**50**, *m/z* 475.3415 [M + H]^+^, C_29_H_46_O_5_), previously characterized from *Sinularia candidula*, nephalsterol B [68] (**52**, *m/z* 431.351 [M + H]^+^, C_28_H_46_O_3_), isomers of hydroxyergostadiene-one [69] (**68**, *m/z* 413.3405 [M + H]^+^, C_28_H_44_O_2_), sarcrasterol [70] (**71**, *m/z* 449.3616 [M + H]^+^, C_28_H_48_O_4_), erectasteroid D [71] (**72**, *m/z* 459.3462 [M + H]^+^, C_29_H_46_O_4_), and hydroxystigmastatrienone [72] (**74**, *m/z* 425.3407 [M + H]^+^, C_29_H_44_O_2_) (Figure 6). The cluster suggested the presence of possible new oxysterol scaffolds with mass differences from the annotated ones of 16 or 17 Da, implying further hydroxylation or hydroperoxylation. 

Lastly, isomers of dihydroxyergostadienone [73] (**75**, *m/z* 429.3359 [M + H]^+^, C_28_H_44_O_3_) were proposed to cluster separately (Figure 6), which could be attributed to the subtle differences in the abundance of the fragment ions as observed with the terpenes metabolites.

### 2.5. Others

Concurrently, the FBMN and the in-silico fragmentation trees proposed by Sirius, assisted in shedding light on overlooked metabolites with potentially undescribed scaffolds. For instance, glycosylated fatty acids, nitrogenated aromatic compounds, and polyketides were proposed in the *Sinularia leptoclados* sample. Former studies reported the occurrence of acylated glycolipids in *Sinularia* species [14]. While marine-derived polyketides have been repeatedly found in various soft coral sources, a growing body of reports point to the coral-associated microorganisms as being the true producers of such chemotypes [74,75]. Additionally, alkaloidal terpenes and *N*-acyl amino acids were proposed in both *Sarcophyton roseum* and *Sarcophyton acutum*. Soft corals are known to produce a variety of *N*-based congeners such as sphingosines, alkaloidal diterpene, purine, and pyrimidine derivatives [15,76,77]. These metabolites play a vital role in protecting the corals against pathogens and environmental stressors. Furthermore, they determine the distribution of the coral as well as the habitat biodiversity [15,76]. In this vein, sporadic reports have dealt with the isolation and characterization of alkaloidal terpenes from soft corals which could be attributed to the typical protocol usually followed, which requires a large amount of living tissue for the isolation of pure compounds and spectroscopic investigation, especially heteronuclear NMR. Yet, *Cladiella*, *Eunicella*, *Sinularia*, and *Lobophytum* were reported in the literature as assemblers of alkaloidal scaffolds [15]. Interestingly, the FBMN proposed the novel presence of alkaloidal terpenes in *Sarcophyton* species for the first time.

Conclusively, the adopted analytical protocol followed in this study proved to be competent and effective for annotating the metabolome of soft corals, allowing the rapid screening of rare and endangered species, and the potential discovery of possible new scaffolds. However, these findings require further chemical investigation for tracing up these potential new scaffolds for their isolation and full characterization. 

## 3. Materials and Methods

### 3.1. Soft Coral Material

The soft coral specimens were collected from the Egyptian Red Sea along the coast of Hurghada, and were identified and authenticated by one of the authors (M.A.A.-H.) (Table 1, Figure S3).

The collected soft coral samples were identified based on morphology, colony color, shape, interior, and sclerites, using established identification keys [78,79,80,81,82,83]. Sclerites or spicules were used for the determination of different soft coral species (Figure S4). Sclerites were obtained by dissolving soft coral tissues in 10% sodium hypochlorite [84]. In addition to the sclerites, taxonomy relied on the presence or absence of siphonozooids among the autozooids (dimorphism), especially to differentiate between the genera *Sarcophyton* and *Sinularia*; and by the number of the autozooids to differentiate between *Sarcophyton* species [79,82], where, colony morphology allows the differentiation and definition of the genus. The coral morphology and the surrounding environment and habitat of the collected soft corals were recorded on an underwater slate.

### 3.2. Chemicals and Reagents

All chemicals for chemical analysis were obtained from Sigma-Aldrich (Merck, Kenilworth, NJ, USA).

### 3.3. Soft Corals Extraction and Sample Preparation for UPLC-MS Analysis

The frozen soft coral specimens (100 g each) were chopped into small pieces and extracted with ethyl acetate (1 L) at room temperature, five times until exhaustion, with respective yields listed in Table 1. The obtained extracts were concentrated under reduced pressure, lyophilized, then kept at −20 °C for further analysis. The lyophilized extracts were prepared for UPLCMS/MS analyses following a previously described protocol [85].

### 3.4. UPLC–HRMS/MS Analysis

The HRMS/MS analysis was carried out on a MaXis 4G instrument (Bruker Daltonics^®^, Bremen, Germany) coupled with an Ultimate 3000 HPLC (Thermo Fisher Scientific^®^, Waltham, MA, USA). A UPLC-method was applied as described in [86]. The separation was carried out on a Nucleoshell 2.7 µm 150 × 2 mm column (Macherey-Nagel^®^, Düren, Germany), and the range for MS acquisition was 50–1800 Daltons (Da). A capillary voltage of 4500 V, nebulizer gas pressure (nitrogen) of 2 (1.6) bar, ion source temperature of 200 °C, dry gas flow of 9 L/min, and spectral rates of 3 Hz for MS^1^ and 10 Hz for MS^2^, were used. For acquiring MS/MS fragmentation, the 10 most intense ions per MS^1^ were selected for subsequent CID, with stepped CID energy applied. The employed parameters for tandem MS were applied as previously detailed [87].

### 3.5. Feature-Based Molecular Networking and Compounds Dereplication

Raw data inspection was performed using Compass Data Analysis 4.4 (Bruker Daltonics^®^). Metaboscape 3.0 (Bruker Daltonics^®^) was utilized for feature detection, grouping, and alignment, employing the T-ReX 3D (Time aligned Region Complete eXtraction) algorithm [40]. Bucketing was performed with an intensity threshold of 10× 10^5^ and a retention time range from 0.5 to 40 min with a restricted mass range *m/z* from 190 to 1800. The produced MGF file and the feature quantification table (CSV file) were used in the feature-based molecular networking (FBMN) following the online workflow in GNPS platform (http://gnps.ucsd.edu (accessed on 1 August 2022)) [23]. The parameters, applied for the construction of the FBMN *via* the GNPS platform, are detailed in Table S2.

Cytoscape version 3.7.1.60 (https://cytoscape.org/, accessed on 15 February 2021) was used for the network visualization. Sirius + CSI:FingerID 4.0.1 was used for the manual putative structures identification [88], assisted by the molecular formula prediction and candidate search with *m/z* tolerance set to 20 ppm connected to online Pubchem and verified through the MarineLit database (https://marinlit.rsc.org/, accessed on 1 August 2022).

## Figures and Tables

**Table 1:** Studied soft coral species and their respective collection sites.

Soft coral Species	Sample Code	Extraction Yield (g)	Location	Coordinates	
				Latitude	Longitude
*Sinularia brassica*	SB	2.8	Northern Safaga	33°56′33.88″ E	26°44′11.92″ N
*Sinularia gardeneri*	SIG	3.1	Northern Safaga	33°56′33.88″ E	26°44′11.92″ N
*Sinularia leptoclados*	SIL	3.4	NIOF-Hurghada	33°46′25.30″ E	27°17′12.07″ N
*Sarcophyton ehrenbergi*	SE	3.8	NIOF-Hurghada	33°46′25.30″ E	27°17′12.07″ N
*Sarcophyton roseum*	SAR	3.7	NIOF-Hurghada	33°46′25.30″ E	27°17′12.07″ N
*Sarcophyton acutum*	SAA	3.5	NIOF-Hurghada	33°46′25.30″ E	27°17′12.07” N
*Litophyton mollis*	LM	3.2	Northern Safaga	33°56′33.88″ E	26°44′11.92″ N
*Cladiella pachyclados*	CLP	2.5/2.4	Marsa Alam	34°53′50.03″ E	25°4′36.26″ N
*Lobophytum pauciflorum*	LOPH	2.2	Marsa Alam	34°53′50.03″ E	25°4′36.26″ N

## Data Availability

The FBMN job on GNPS can be accessed at https://gnps.ucsd.edu/ProteoSAFe/status.jsp?task=5c2975ad4ac541f3bf96c564b20d809c (accessed on 1 August 2022).

## Supplementary Materials

The following supporting information can be downloaded at: https://www.mdpi.com/article/10.3390/md20100630/s1, Figure S1: The base peak chromatograms of the studied soft corals extracts in positive ionization mode; Figure S2: Comparative MS/MS spectra of compounds **9**, **41**, **62** justifying their de-clustering in the FBMN; Figure S3: Google Earth map showing the location of collection sites; Figure S4: The endoskeleton of the most common genera: (A) *Sinularia* sp., (B) *Sarcophyton* sp.; Table S1: Putative compound assignment of the studied soft coral specimens as revealed by UPLC-HRMS/MS analysis; Table S2: Parameters used for the construction of the FBMN via the GNPS platform.
