# Supplementary material for: Molecular Network Guided Cataloging of the Secondary Metabolome of Selected Egyptian Red Sea Soft Corals
Source: Mar Drugs. 2022 Oct 1;20(10):630. doi: 10.3390/md20100630 (PMC9604675; doi:10.3390/md20100630)
Supplement: Supplementary file 1 [file marinedrugs-20-00630-s001.zip › marinedrugs-1896352-supplementary.pdf]

Supplementary Data

Molecular Network Guided Cataloging of the Secondary Metabolome of Selected Egyptian Red Sea Soft Corals

Nesrine M. Hegazi [1], Tarik A. Mohamed [2], Hamada H. Saad [3], Montaser A. Al-Hammady [4], Taha A. Hussien [5], Mohamed-Elamir F. Hegazy [2] and Harald Gross [3,*]

[1] Department of Phytochemistry and Plant Systematics, National Research Centre, 33 El-Bohouth St., Dokki, Giza 12622, Egypt
[2] Chemistry of Medicinal Plants Department, National Research Centre, 33 El-Bohouth St., Dokki, Giza 12622, Egypt
[3] Department of Pharmaceutical Biology, Pharmaceutical Institute, University of Tübingen, Auf der Morgenstelle 8, 72076 Tübingen, Germany
[4] National Institute of Oceanography and Fisheries (NIOF), Cairo 11516, Egypt
[5] Pharmacognosy Department, Faculty of Pharmacy, Sphinx University, New Assiut City, Assiut 10, Egypt
* Correspondence: harald.gross@uni-tuebingen.de

Figure S1: The base peak chromatograms of the studied soft corals extracts in the positive ionization mode.

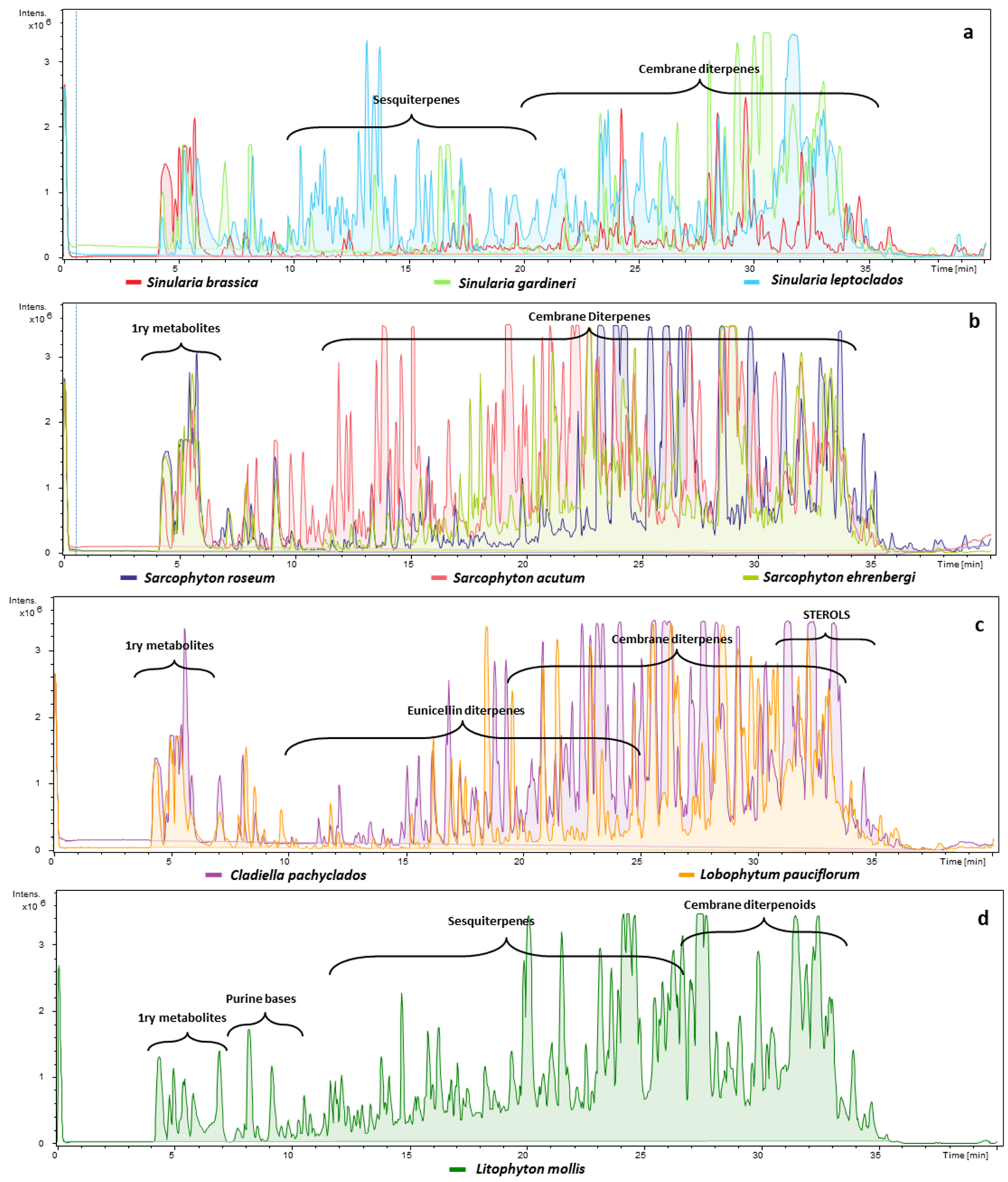

Figure S2: Comparative MS/MS spectra of compounds 9, 41, 62 justifying their de-clustering in the constructed FBMN.

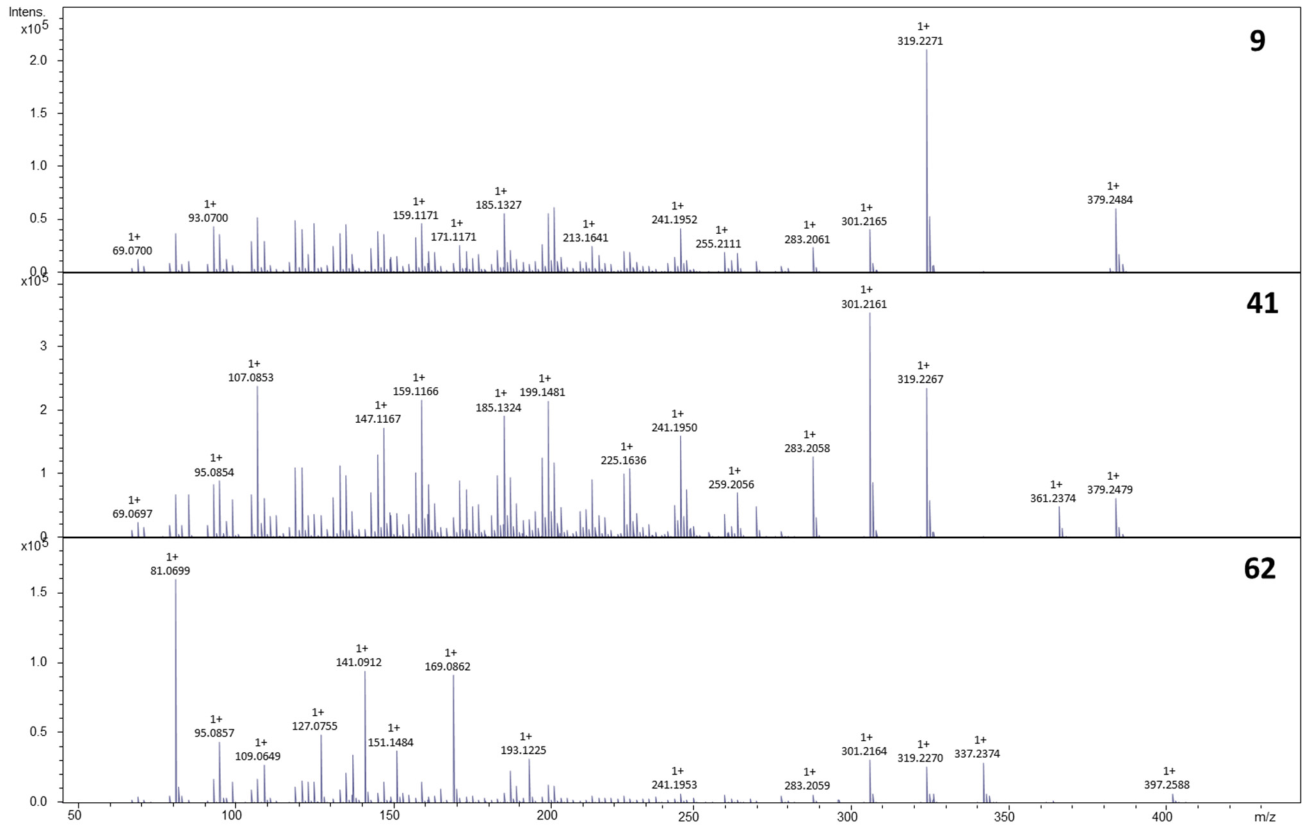

Figure S3: Google earth map showing the location of collection sites

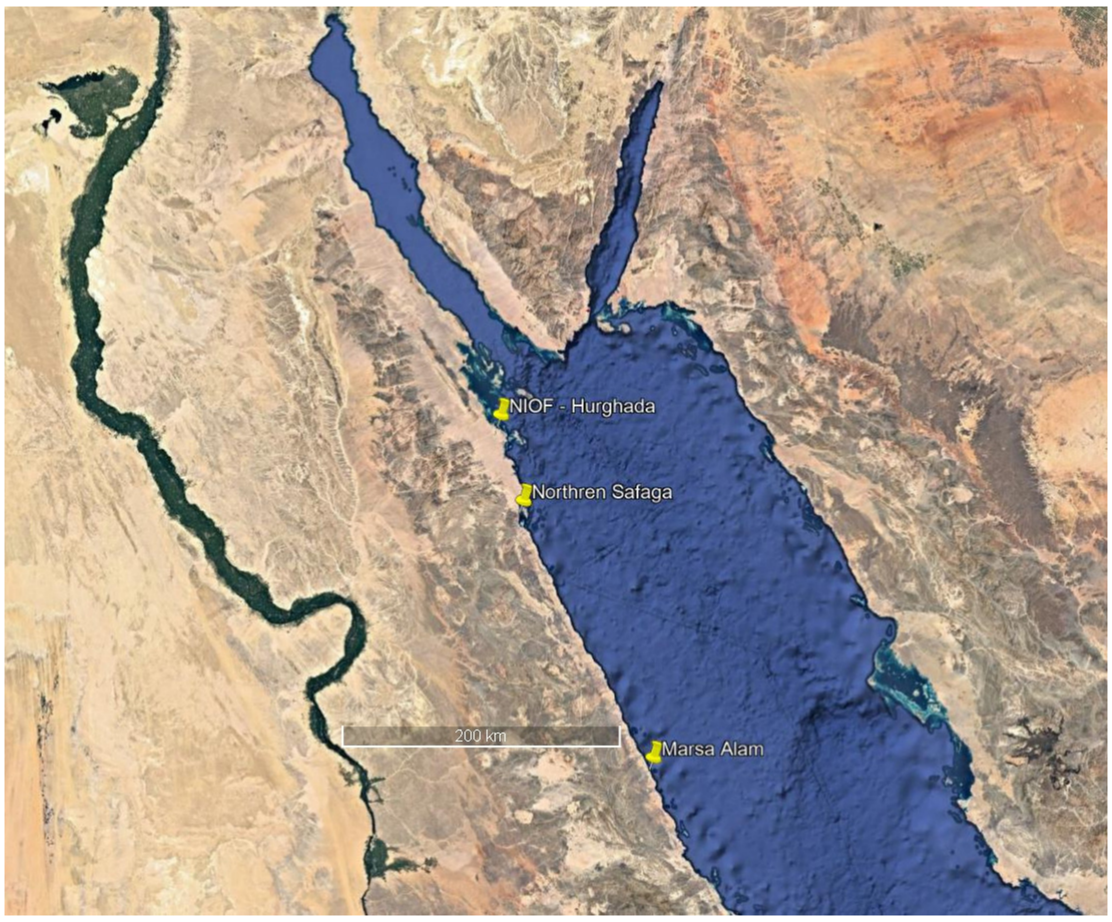

Figure S4: The endoskeleton of the most common genera; A) *Sinularia* sp., B) *Sarcophyton* sp.

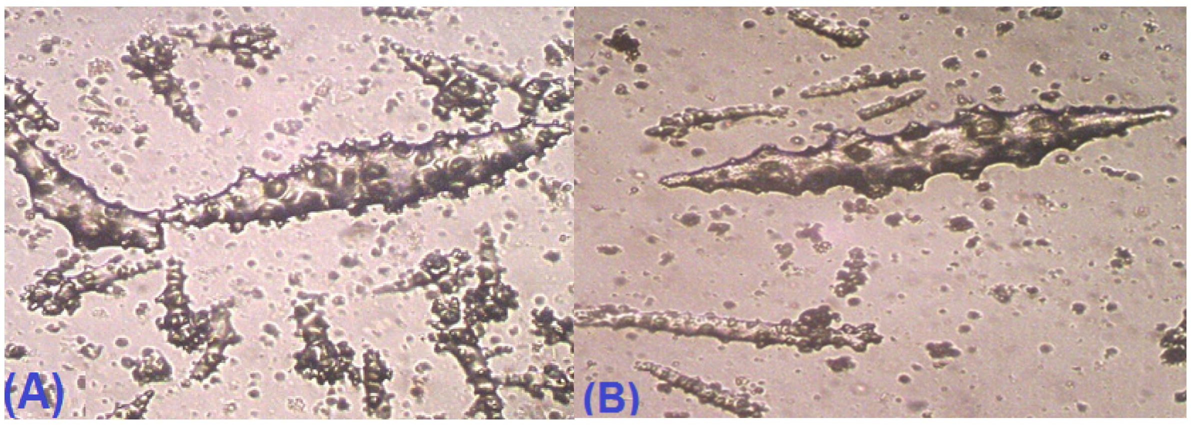

Table S1: Compound assignment of the studied soft coral specimens as revealed by UPLC-HRMS/MS analysis. Abbreviations: SB: *Sinularia brassica*, SIG: *Sinularia gardineri*, SIL: *Sinularia leptoclados*, SE: *Sarcophyton ehrenbergi*, SAR: *Sarcophyton roseum*, SAA: *Sarcophyton acutum*, LM: *Litophyton mollis*, CLP: *Cladiella pachyclados*, LOPH: *Lobophytum pauciflorum*. -: absent, tr: traces, +: present, ++: abundant, +++: major.

Nr.	Compound class	t_R	$[M+H]^+$	Fragments	Molecular formula (error in ppm)	Proposed structure	Abundance in the selected soft coral specimens									References
							SB	SIG	SIL	SE	SAR	SAA	LM	CLP	LOPH	
1.	Eunicellin* diterpenoids	11.32	355.2484	319.2277<br>301.2164<br>283.2062<br>273.2218<br>259.2063<br>249.1856<br>241.1954<br>229.1596<br>213.164<br>201.1642<br>185.133<br>161.1318<br>153.0914<br>147.1169<br>135.1170<br>127.0754<br>109.1015<br>95.0492<br>81.0699<br>69.0697	$C_{20}H_{34}O_5$ (-1.4)	Sclerophytin D	tr	tr	tr	tr	tr	tr	tr	+++	tr	[1]
2.	Sphingoid bases	11.91	296.2583	278.2482<br>233.1906<br>215.1798<br>205.1588<br>189.1640<br>173.1328<br>159.1171<br>147.1171<br>133.1012<br>119.0857<br>105.0700<br>81.0700<br>58.0650	$C_{18}H_{33}NO_2$ (0.4)	Sphingatrienine	-	-	-	-	-	+	+++	-	-	[2]
3.	β-caryophyllene-sesquiterpene	13.73	219.1742	186.1408<br>173.1328<br>159.1171<br>145.1015<br>131.0858<br>119.0858<br>105.0700<br>93.0700<br>85.0649<br>69.0698	$C_{15}H_{23}O$	Buddledin C/D	-	-	-	-	-	+	+++	-	-	[3]

4.	Asteriscane sesquiterpenes	14.51	269.1748	233.1541<br>215.1435<br>197.1325<br>191.1071<br>173.0966<br>145.1015<br>133.1015<br>109.0648<br>93.0701<br>81.0699<br>71.0494	$C_{15}H_{24}O_4$<br>(-7.0)	Capillosanane J	+	+	-	+	-	+++	+++	+	+	[4]
5.	Cembrane diterpenoids	15.3	333.2067	315.1965<br>297.1847<br>279.1751<br>239.1432<br>225.1651<br>215.1435<br>197.1325<br>183.1166<br>175.112<br>157.1012<br>145.1011<br>135.0807<br>119.0856<br>105.0701<br>93.07<br>81.0698<br>69.07	$C_{20}H_{28}O_4$<br>(-2.0)	Hexahydro-10-hydroxy-3,6,10,14-tetramethylcyclotetradeca[*b*]furan-2,11(4*H*,8*H*)-dione isomer I	+	-	-	+++	+	-	-	-	-	[5]
6.	Cembrane diterpenoids	16.54	367.2111	348.2903<br>331.1545<br>315.1958<br>297.1854<br>279.1748<br>269.1904<br>251.1801<br>239.1434<br>225.1276<br>213.1276<br>197.1328<br>183.1168<br>171.1171<br>157.1014<br>145.1014<br>131.0856<br>119.0857<br>105.07<br>93.0699<br>81.0699<br>67.0543	$C_{20}H_{30}O_6$<br>(1.2)	Sinularolide A	tr	-	tr	+		+++	-	-	tr	[6]
7.	Guaiane sesquiterpenes	16.59	277.1775	260.2377<br>215.1797<br>201.1638<br>187.1481<br>173.1324	$C_{17}H_{24}O_3$ (8.6)	Spiro[azulene-5(1H),2'-oxiran]-4-ol,<br>3a,4,6,7,8,8a-hexahydro-3,3',3'-trimethyl-8-methylene-, 4-acetate	+	+	+++	-	-	-	++	+	-	[7]

				159.1169<br>145.1014<br>131.0856<br>119.0859<br>105.0701<br>93.0697<br>81.0699<br>69.0698<br>58.0649											
8.	Eudesmane sesquiterpenes	16.60	219.1744	201.1641<br>191.1435<br>175.1485<br>161.1329<br>145.1015<br>121.0650<br>105.0700<br>95.0857<br>81.0700	$C_{15}H_{22}O$ (-0.8)	2(1H)-Naphthalenone, 3,4,6,7,8,8a-hexahydro-5,8a-dimethyl-3-(1-methylethylidene) isomer I	-	++	+++	+	-	-	++	++	+	[8]
9.	Eunicellin diterpenoids	16.78	379.2476	319.2271<br>301.2165<br>283.2061<br>241.1952<br>213.1641<br>201.1641<br>185.1327<br>159.1171<br>145.1014<br>135.1171<br>125.0963<br>107.0856<br>93.0700<br>81.0699<br>69.0700	$C_{22}H_{34}O_5$ (-2.1)	Hirsutalin G	-	+	+	+	+	+	-	+++	+	[9]
10.	Eudesmane sesquiterpenes	17.37	219.1739	201.1641<br>191.1435<br>175.1485<br>161.1329<br>145.1015<br>121.0650<br>105.0700<br>95.0857<br>81.0700	$C_{15}H_{22}O$ (1.9)	2(1H)-Naphthalenone, 3,4,6,7,8,8a-hexahydro-5,8a-dimethyl-3-(1-methylethylidene) isomer II	-	-	+	-	-	-	+	-	-	[8]
11.	Cembrane diterpenoids	18.00	349.2002	330.2792<br>313.1799<br>295.1694<br>267.1746<br>253.123<br>243.1385<br>225.1278<br>209.1328<br>197.1327<br>183.1168<br>173.0963	$C_{19}H_{24}O_6$ (-7.0)	Gyrosanolide A	++	+	+	-	+	+	+	-	+++	[10]

				163.0754<br>149.0961<br>131.0857<br>119.0857<br>105.07<br>93.0699<br>85.0649<br>67.0542												
12.	Asteriscane sesquiterpenoids	18.49	235.1690	219.1744<br>201.1641<br>189.1640<br>177.1640<br>161.1327<br>151.1120<br>145.1014<br>133.1013<br>119.0857<br>105.0700<br>95.0857<br>81.0699	$C_{15}H_{22}O_2$ (0.3)	Capillosanane D	-	-	-	-	-	-	-	-	-	[4]
13.	Cembrane diterpenoids	18.82	331.1897	315.196<br>297.1858<br>267.1747<br>239.1430<br>225.1276<br>209.1330<br>197.1330<br>187.1119<br>163.0761<br>145.1016<br>135.0808<br>119.0857<br>109.0648<br>93.0699<br>81.0698<br>69.0698	$C_{20}H_{26}O_4$ (2.0)	Sarcostolide isomer I	++	+	+	++	+	+++	-	+	+	[11]
14.	Cembrane diterpenoids	18.86	319.2263	301.2163<br>283.2055<br>257.1901<br>241.1589<br>223.1483<br>215.1432<br>197.1326<br>185.1328<br>171.1171<br>159.1167<br>147.1173<br>133.1011<br>119.0859<br>107.0856<br>93.0698<br>81.07	$C_{20}H_{30}O_3$ (1.4)	14β-Hydroxy-2-epi-16-deoxysarcophine	-	+	+	+	+	+	-	+	+	[12]
15.	Guaiane sesquiterpenes	19.22	199.1478	182.1088<br>169.1006	$C_{15}H_{18}$ (1.7)	Cuteazul	tr	-	-	-	tr	-	+++	-	tr	[13]

				157.1011<br>145.1012<br>131.0853<br>119.0856<br>105.0697<br>93.0697<br>81.0698<br>69.0697												
16.	Eunicellin diterpenoids	19.51	421.2581	319.2273<br>301.2166<br>283.2061<br>265.1956<br>255.211<br>241.159<br>223.1484<br>215.1797<br>199.1483<br>185.1326<br>173.1326<br>157.1012<br>147.1169<br>131.0855<br>119.0856<br>105.0699<br>95.0491<br>81.0698<br>69.0698	$C_{24}H_{36}O_6$ (0.8)	Astrogorgin B isomer I	-	-	tr	-	tr	-	-	-	+++	[14]
17.	Cembrane diterpenoids	19.55	351.2160	333.2058<br>315.1956<br>297.1856<br>279.1744<br>267.1746<br>255.1381<br>239.1433<br>225.1275<br>213.1273<br>197.1327<br>187.1119<br>173.0963<br>161.0962<br>149.0962<br>145.1013<br>135.0806<br>105.0699<br>93.0699<br>81.0699<br>67.0541	$C_{20}H_{30}O_5$ (1.5)	Sarcoehrenbergilid C	+	-	-	+++	-	++	+	-	-	[15]
18.	β-caryophyllene-sesquiterpene	19.58	219.1741	201.1643<br>161.1327<br>145.1014<br>131.0857<br>119.0857<br>105.0700<br>93.0700	$C_{15}H_{23}O$ (0.8)	Buddledin C/D	-	+	+	+	-	+	+++	-	+	[3]

				81.0699<br>69.0699											
19.	Asteriscane sesquiterpenoids	19.84	235.1686	217.1588<br>193.1589<br>189.1638<br>159.1168<br>149.1325<br>119.0856<br>107.0855<br>93.0699<br>85.0648<br>71.0490	$C_{15}H_{22}O_2$ (0.3)	Capillosanane M	-	-	+	-	-	-	+++	-	+	[4]
20.	Eudesmane sesquiterpenoids	19.89	253.1791	217.1588<br>193.1589<br>175.1481<br>159.1168<br>147.1168<br>135.1168<br>119.0855<br>113.0597<br>107.0855<br>93.0698<br>85.0647<br>81.0697<br>71.049	$C_{15}H_{24}O_3$ (2.7)	Oxoeudesmendiol isomer I	tr	tr	+	-	tr	-	+++	tr	Tr	[16]
21.	Calamenene sesquiterpene	20.01	203.1789	161.1325<br>147.117<br>133.1013<br>119.0856<br>105.0699<br>95.0856<br>81.0699<br>69.0698	$C_{15}H_{22}$ (1.7)	Calamenene	-	-	-	-	-	-	+++	-	tr	[17]
22.	β-caryophyllene-sesquiterpene	20.1	221.1893	203.1794<br>177.1638<br>163.1481<br>147.1167<br>135.1167<br>119.0854<br>107.0854<br>95.0854<br>81.0697<br>69.0697	$C_{15}H_{24}O$ (2.9)	Suberosol C	-	+	-	-	-	-	+++	-	-	[3]
23.	Cembrane diterpenoids	20.36	381.2259	329.211<br>315.1953<br>297.185<br>279.1747<br>269.1902<br>251.1796<br>237.1275<br>225.1275<br>209.1325<br>197.1325<br>183.1171	$C_{21}H_{32}O_6$ (1.9)	Briaviodiol A isomer I	+	+	+	+	+	+++	-	+	-	[18]

				171.1167<br>157.1013<br>145.1013<br>133.1013<br>119.0856<br>107.0855<br>93.07<br>81.0698<br>67.0541												
24.	Cembrane diterpenoids	20.74	331.1894	315.1955<br>297.1854<br>279.1745<br>251.1796<br>227.1432<br>209.1326<br>195.1167<br>183.117<br>175.1117<br>161.0961<br>145.1012<br>135.0806<br>119.0857<br>105.0699<br>95.0491<br>81.0698<br>69.0698	$C_{20}H_{26}O_4$ (2.9)	Sarcostolide isomer II	++	+	-	-	-	+++	-	-	-	[11]
25.	Eunicellin diterpenoids	20.85	381.2627	321.2426<br>303.2319<br>285.2215<br>225.1639<br>201.164<br>163.1482<br>153.091<br>137.1325<br>127.0754<br>107.0854<br>95.0491<br>81.0698	$C_{22}H_{36}O_5$ (2.3)	Sclerophytin B	-	-	-	-	-	-	-	+++	-	[19]
26.	Cembrane diterpenoids	21.02	287.2367	269.2266<br>241.1957<br>229.1953<br>213.1637<br>199.1482<br>185.1326<br>173.1326<br>159.117<br>147.1168<br>133.1014<br>119.0857<br>107.0856<br>95.0856<br>81.07<br>69.0699	$C_{20}H_{30}O$ (0.7)	Sinularone A	-	+	+	-	-	+	++	-	-	[20]

27.	Eunicellin diterpenoids	21.44	467.2999	337.2375<br>319.2269<br>301.2163<br>273.2215<br>223.1483<br>213.1638<br>199.1482<br>187.1481<br>161.1323<br>147.1166<br>127.0751<br>107.0853	$C_{26}H_{42}O_7$ (1.0)	Pachycladin B	-	-	-	-	-	-	-	-	+++	[21]
28.	Cembrane diterpenoids	21.83	395.2423	377.232<br>357.2036<br>337.2371<br>317.2114<br>299.2007<br>281.1902<br>271.2058<br>257.1906<br>239.143<br>229.1592<br>215.1432<br>201.1274<br>185.1325<br>171.1174<br>157.1009<br>145.1012<br>133.1013<br>121.1014<br>109.0649<br>95.0856<br>81.0699<br>69.0699	$C_{22}H_{34}O_6$ (1.3)	Sarcophytonolide Q<br><br>C	tr	-	-	tr	-	tr	-	tr	-	[22]
29.	Cembrane diterpenoids	22.37	423.2375	389.1945<br>345.2065<br>327.1968<br>313.1802<br>295.1691<br>285.1852<br>267.1749<br>253.1587<br>225.1638<br>211.112<br>197.1326<br>183.1168<br>163.1118<br>149.0958<br>137.0599<br>121.0651<br>107.0492<br>93.07<br>81.0698<br>69.0697	$C_{23}H_{34}O_7$<br>(0.6)	Durumolide O	+++	-	tr	-	tr	tr	-	-	tr	[23]

30.	Cembrane diterpenoids	22.38	349.1994	331.1904<br>313.1795<br>297.1846<br>279.1743<br>269.1897<br>251.1791<br>237.1271<br>225.1272<br>209.1322<br>197.1323<br>183.1163<br>171.1165<br>157.1009<br>145.1010<br>133.1010<br>119.0854<br>107.0853<br>93.0697<br>81.0697<br>69.0697	$C_{20}H_{28}O_5$ (3.5)	Sarcoconvolutum D	+	+	+	+++	+	-	+	-	+	[24]
31.	Cembrane diterpenoids	22.49	381.2259	329.211<br>315.1953<br>297.185<br>279.1747<br>269.1902<br>251.1796<br>237.1275<br>225.1275<br>209.1325<br>197.1325<br>183.1171<br>171.1167<br>157.1013<br>145.1013<br>133.1013<br>119.0856<br>107.0855<br>93.07<br>81.0698<br>67.0541	$C_{21}H_{32}O_6$ (3.3)	Briaviodiol A isomer II	+	+	+	+	+	+++	-	+	-	[18]
32.	Cembrane diterpenoids	22.67	331.1894	313.1798<br>295.1688<br>285.1851<br>277.1589<br>267.1741<br>255.1377<br>237.1273<br>227.1429<br>209.1323<br>197.1322<br>183.1167<br>175.1114<br>157.1009<br>145.1012<br>131.0856	$C_{20}H_{26}O_4$ (2.9)	Sarcostolide isomer III	++	+	+	++	+	+++	-	+	-	[11]

				119.0855<br>107.0855<br>93.0699<br>81.0696<br>67.0541												
33.	Eunicellin diterpenoids	22.8	439.2687	337.2379<br>319.2272<br>301.2165<br>273.2217<br>255.2111<br>241.1591<br>223.1484<br>213.1641<br>199.1484<br>187.1483<br>173.1326<br>161.1326<br>147.1168<br>127.0754<br>119.0856<br>105.0699<br>95.0855<br>83.0491<br>69.0698	$C_{24}H_{38}O_7$ (0.8)	Klysimplexin E	-	-	-	-	-	-	-	-	+++	[38]
34.	Eunicellin diterpenoids	22.88	449.2892	319.2271<br>301.2165<br>283.2059<br>265.1954<br>255.2110<br>241.1588<br>223.1483<br>215.1796<br>205.1588<br>199.1482<br>185.1325<br>173.1324<br>165.0909<br>147.1167<br>137.0961<br>131.0854<br>121.1011<br>105.0698<br>95.049	$C_{26}H_{40}O_6$ (1.2)	Litophynol A acetate isomer I	tr	Tr	tr	tr	tr	tr	tr	-	+++	[25]
35.	Cembrane diterpenoids	22.91	349.1998	331.1901<br>313.1799<br>297.1851<br>279.1745<br>269.1899<br>251.1796<br>237.1272<br>225.1272<br>213.1271<br>209.1324<br>197.1324	$C_{20}H_{28}O_5$ (1.1)	Hydroperoxysarcophine isomer I	+	+	+	+++	+	+	+	+++	+	[26]<br>[27]

				183.1163<br>171.1167<br>157.101<br>145.1012<br>133.1012<br>119.0855<br>107.0854<br>93.0698<br>81.0698<br>69.0698												
36.	Cembrane diterpenoids	23.41	349.1998	297.1849<br>279.1743<br>269.1901<br>251.1794<br>225.1273<br>209.1324<br>197.1324<br>183.1165<br>171.1166<br>157.1010<br>145.1011<br>119.0854<br>107.0854<br>93.0698<br>81.0697<br>69.0697	$C_{20}H_{28}O_5$ (1.7)	Hydroperoxysarcophine isomer II	-	-	-	-	-	+++	-	+	-	[26]<br>[27]
37.	Cembrane diterpenoids	23.53	349.1994	331.1904<br>313.1795<br>297.1846<br>279.1743<br>269.1897<br>251.1791<br>237.1271<br>225.1272<br>209.1322<br>197.1323<br>183.1163<br>171.1165<br>157.1009<br>145.1010<br>133.1010<br>119.0854<br>107.0853<br>93.0697<br>81.0697<br>67.054	$C_{20}H_{28}O_5$ (3.5)	Hydroperoxysarcophine isomer III	+	+	+	+++	+	-	+	-	-	[26]<br>[27]
38.	Dolabellane Diterpenoids	23.74	287.237	269.2268<br>241.195<br>227.1795<br>213.1636<br>199.1485<br>185.1326<br>171.1177<br>157.1018	$C_{20}H_{30}O$ (-0.1)	Eduenone	tr	tr	-	tr	+++	tr	+	tr	tr	[28]

				145.1012<br>133.1013<br>121.1014<br>107.0857<br>95.0856<br>81.0698<br>69.0699												
39.	Cembrane diterpenoids	23.88	305.2466	287.237<br>269.2264<br>243.2108<br>227.1795<br>213.1638<br>199.1481<br>185.1325<br>171.1168<br>159.1167<br>147.1167<br>133.1012<br>121.1011<br>107.0855<br>95.0855<br>81.0698<br>69.0697	$C_{20}H_{32}O_2$ (2.7)	Sarglaucol	+	-	-	-	+++	+	+	-	-	[29]
40.	Asteriscane sesquiterpenoids	23.91	253.1790	187.148<br>175.148<br>159.1168<br>147.1168<br>135.1168<br>119.0855<br>113.0597<br>107.0855<br>93.0698<br>85.0646<br>81.0698<br>71.049	$C_{15}H_{24}O_3$ (3.2)	Oxoeudesmendiol isomer II	tr	-	-	-	-	-	+++	tr	tr	[4]
41.	Eunicellin diterpenoids	24.09	379.2474	361.2374<br>319.2267<br>301.2161<br>283.2058<br>265.1951<br>259.2056<br>241.195<br>225.1636<br>199.1481<br>185.1324<br>159.1166<br>147.1167<br>133.1011<br>121.1011<br>107.0853<br>95.0854<br>69.0697	$C_{22}H_{34}O_5$ (0.9)	Klymollin S	tr	tr	-	-	-	-	-	+++	tr	[30]
42.	Cembrane diterpenoids	24.11	317.2103	299.2010<br>281.1900	$C_{20}H_{28}O_3$ (-3.3)	Ehrenbergol D	-	-	-	-	-	-	-	+++	++	[31]

				259.1694<br>241.159<br>229.1588<br>223.1483<br>213.1639<br>201.1275<br>183.1169<br>173.1325<br>161.0963<br>145.1014<br>133.1013<br>119.0856<br>105.0699<br>93.0700<br>81.0699<br>67.0541												
43.	Cembrane diterpenoids	24.19	323.2572	305.2477<br>287.2372<br>269.2265<br>259.2059<br>227.1794<br>213.1638<br>199.1483<br>173.1325<br>159.1168<br>147.1167<br>133.1011<br>121.1011<br>107.0854<br>95.0854<br>81.0697<br>69.0697	$C_{20}H_{34}O_3$ (2.7)	Epoxycembradienediol	+	+	+	-	-	-	-	+	+	[32]
44.	Cembrane diterpenoids	24.7	287.2365	269.2262<br>245.1901<br>227.1790<br>213.1639<br>185.1326<br>159.1164<br>145.1011<br>121.1011<br>107.0855<br>93.0698<br>81.0698<br>69.0698	$C_{20}H_{30}O$ (0.0)	Sarcophytonin A / Deoxysarcophytoxide	++	++	++	+++	-	+++	-	-	-	[33]
45.	Eunicellin diterpenoids	24.73	423.2748	363.2535<br>345.243<br>321.2429<br>303.2322<br>285.2216<br>267.2111<br>243.1747<br>225.164<br>215.1797<br>201.164	$C_{24}H_{38}O_6$ (-1.7)	Oxylitophynol	tr	-	tr	+ tr	-	tr	tr	+++	+++	[25]

				187.1484<br>163.1482<br>141.0911<br>127.0754<br>107.0856<br>95.0856<br>81.0698<br>69.0699												
46.	Cembrane diterpenoids	24.81	335.2208	317.211<br>299.2004<br>281.1901<br>275.2003<br>259.169<br>241.1587<br>219.1382<br>211.1481<br>201.1276<br>189.1275<br>175.1117<br>161.0962<br>149.0961<br>133.1012<br>119.0855<br>105.0698<br>93.0698<br>81.0698<br>67.054	$C_{20}H_{30}O_4$ (1.5)	Dihydroxydeepoxy-sarcophine	+	-	-	+++	++	++	+	-	+	[15]
47.	Asteriscane sesquiterpenoids	24.83	233.1528	199.1481<br>189.1637<br>175.1117<br>157.1012<br>145.1012<br>133.1012<br>119.0855<br>105.0699<br>93.0698<br>81.0698<br>71.049	$C_{15}H_{20}O_2$ (-5.2)	Capillosanane F	+	-	++	-	-	+++	+++	-	-	[4]
48.	Cembrane diterpenoids	24.87	365.2316	333.2059<br>315.1958<br>297.1854<br>287.2009<br>279.1747<br>269.1902<br>251.1799<br>237.1278<br>227.1435<br>209.1327<br>191.1069<br>175.1121<br>161.0963<br>145.1013<br>135.0806<br>121.1013	$C_{21}H_{32}O_5$ (1.4)	Durumolide Q	++	+	+	++	-	+++	+	+	-	[23]

				107.0857<br>95.0492<br>85.0647<br>69.0700											
49.	Eunicellin diterpenoids	25.15	381.2633	321.2424<br>303.2322<br>285.2216<br>267.2114<br>225.1641<br>199.1487<br>189.1641<br>163.1486<br>153.0912<br>137.1326<br>127.0753<br>107.0858<br>81.0698	$C_{22}H_{36}O_5$<br>(0.45)	Sclerophytin E isomer I	tr	Tr	tr	tr	Tr	tr	tr	+++	+++	[34]
50.	Sterols	25.20	475.3415	457.3329<br>439.3208<br>336.3261<br>319.2632<br>235.132<br>205.1227<br>177.0908<br>165.0912<br>135.1165<br>123.1169<br>109.1015<br>95.0855<br>81.07<br>71.0492	$C_{29}H_{46}O_5$<br>(0.7)	5,8-Epidioxy-3,25-dihydroxy-4-methylergost-9(11)-en-2-one	tr	-	tr	tr	tr	-	tr	-	tr	[35]
51.	Cembrane diterpenoids	25.34	339.252	303.2322<br>285.2217<br>269.2266<br>243.1746<br>227.1797<br>213.1635<br>201.1637<br>187.1482<br>173.1325<br>159.1169<br>147.117<br>133.1012<br>121.1013<br>107.0856<br>95.0856<br>81.0698	$C_{20}H_{34}O_4$<br>(3.0)	Sinulariol B	+	+	+	+	+++	-	-	+	+	[36]
52.	Sterols	25.41	431.351	395.331<br>377.3204<br>365.32<br>309.2581<br>271.2058<br>253.1952<br>239.1795	$C_{28}H_{46}O_3$<br>(2.2)	Nephalsterol B	-	+	+	+	+	-	-	-	-	[37]

				227.1794<br>213.1638<br>199.1484<br>185.1326<br>173.1326<br>159.117<br>147.117<br>141.1275<br>123.117<br>109.1013<br>95.0857<br>81.0699<br>69.0698												
53.	Eunicellin diterpenoids	25.45	381.2633	321.2429<br>303.2325<br>285.2214<br>225.1642<br>199.1479<br>163.1483<br>153.0913<br>137.1325<br>127.0755<br>107.0856<br>95.0492<br>81.0698	$C_{22}H_{36}O_5$ (0.8)	Sclerophytin E isomer II	tr	tr	tr	tr	Tr	tr	tr	+++	+++	[34]
54.	Eunicellin diterpenoids	26.25	467.2995	337.2375<br>319.2269<br>301.2163<br>283.2058<br>223.1481<br>213.1638<br>199.1481<br>187.148<br>161.1322<br>147.1165<br>127.075<br>107.0853<br>95.0853<br>83.0488	$C_{26}H_{42}O_7$ (1.8)	Klysimplexin C	--	--	--	--	--	--	--	--	+++	[38]
55.	Cembrane diterpenoids	26.3	391.211	355.1891<br>331.1906<br>315.1959<br>299.1648<br>281.1539<br>271.1697<br>253.1591<br>243.1382<br>229.1226<br>215.1071<br>204.1143<br>189.0914<br>177.0913<br>165.0912<br>157.1017	$C_{22}H_{30}O_6$ (1.3)	Sarcocrassocolide A	+	+	-	++	++	-	-	-	-	[39]

				145.1013<br>133.1015<br>119.0856<br>107.0857<br>95.0855<br>81.0699<br>69.0697												
56.	Eunicellin diterpenoids	26.35	379.2471	319.2267<br>301.2162<br>283.2058<br>273.2215<br>255.2108<br>213.1638<br>199.1481<br>185.1324<br>159.1168<br>149.1324<br>135.1168<br>121.1011<br>107.0854<br>95.0855<br>81.0698<br>69.0697	$C_{20}H_{35}O_5$<br>(-0.5)	Astrogorgin L	-	-	-	-	-	-	-	+++	-	[14]
57.	Eunicellin diterpenoids	26.48	421.2577	319.2268<br>301.2163<br>265.1951<br>223.1481<br>215.1794<br>199.1481<br>185.1325<br>173.1323<br>157.101<br>147.1167<br>131.0853<br>119.0854<br>107.0854<br>95.0489	$C_{24}H_{36}O_6$<br>(1.8)	Astrogorgin B isomer II	-	-	tr	-	tr	-	-	-	+++	[14]
58.	Cembrane diterpenoids	26.64	287.236	269.2267<br>259.2429<br>241.1954<br>227.1794<br>213.164<br>199.1485<br>185.1326<br>173.1325<br>159.1171<br>147.1169<br>133.1013<br>121.1012<br>109.1014<br>95.0857<br>81.0699<br>81.0699	$C_{20}H_{30}O_2$<br>(1.8)	Sarcophytoxide	+	-	-	-	+++	-	-	-	-	[40]

59.	Cembrane diterpenoids	26.70	317.2105	299.2009<br>281.1905<br>269.1901<br>251.1799<br>241.1591<br>225.1640<br>211.1485<br>197.1328<br>183.1168<br>175.1118<br>161.0965<br>145.1015<br>133.1013<br>119.0855<br>107.0857<br>93.0699<br>81.0699<br>69.0699	$C_{20}H_{28}O_3$<br>(-4.1)	Isosarcophinone	+	-	+	+++	+	-	-	-	+	[41]
60.	Cembrane diterpenoids	26.99	305.2465	287.2369<br>269.2262<br>259.2422<br>245.1899<br>227.1794<br>213.1638<br>201.1637<br>175.1479<br>149.1323<br>135.1167<br>121.1011<br>107.0854<br>95.0854<br>81.0697<br>69.0697	$C_{20}H_{32}O_2$<br>(3.2)	Sarcophytol P	+	+	-	+	+++	+	+	-	+	[42]<br>[43]
61.	Cembrane diterpenoids	27.19	321.2419	303.2324<br>285.2217<br>267.2105<br>257.2264<br>243.1739<br>225.1646<br>215.1796<br>199.1480<br>187.1486<br>173.1332<br>163.1484<br>147.117<br>135.1171<br>121.1016<br>107.0857<br>93.0700<br>81.0700	$C_{20}H_{32}O_3$<br>(1.4)	Dihydroxydeepoxy-sarcophytoxide<br><br>Sachropytonin C isomer	-	tr	tr	-	-	tr	-	+++	+	[44]
62.	Eunicellin diterpenoids	27.25	397.2579	337.2375<br>319.2273<br>301.2164<br>241.1952	$C_{22}H_{36}O_6$<br>(1.7)	Sclerophytin C	tr	tr	tr	tr	0	-	tr	+++	+++	[34]

				193.1226<br>169.0861<br>141.0912<br>127.0756<br>109.0649<br>95.0857<br>81.0699												
63.	Cembrane diterpenoids	27.28	349.2365	331.1905<br>299.1652<br>271.1695<br>257.1541<br>239.1434<br>211.1486<br>197.1326<br>187.1119<br>161.0964<br>151.1119<br>145.1015<br>133.1013<br>119.0856<br>107.0857<br>93.0699<br>81.0699<br>67.0542	$C_{21}H_{32}O_4$ (2.3)	Stellatumonin B	+	+	+	+++	+++	-	+	-	+	[45]
64.	Eunicellin diterpenoids	27.90	337.2368	319.2268<br>301.2164<br>273.2212<br>255.2108<br>233.1533<br>193.1226<br>169.0862<br>141.0913<br>109.1014<br>93.0700<br>81.0699	$C_{20}H_{32}O_4$ (1.4)	Klymollin Y	+	+	+	-	++	+	-	+++	+	[46]
65.	Cembrane diterpenoids	28.02	367.211	349.2009<br>317.1749<br>299.1644<br>289.1802<br>271.1695<br>257.1539<br>247.1694<br>237.1122<br>229.159<br>219.1018<br>195.1017<br>177.091<br>167.0702<br>151.1117<br>139.0754<br>123.1169<br>109.1012<br>95.0855<br>81.0697	$C_{20}H_{30}O_6$ (1.3)	Sinulaparvalide A	+	-	tr	+	+++	-	tr	tr	tr	[47]

				69.0699												
66.	Eunicellin diterpenoids	28.09	447.2743	373.2737<br>321.243<br>303.2321<br>285.2215<br>267.2111<br>257.2266<br>239.1792<br>225.1641<br>211.1483<br>197.1329<br>187.1483<br>173.1326<br>163.1482<br>147.1171<br>141.0911<br>127.0755<br>107.0856<br>93.0699<br>81.0698<br>71.0491	$C_{26}H_{38}O_6$<br>(-0.4)	2-(Acetyloxy)tetradeca-hydro-6-methyl-1,10-bis(methylene)-4-(1-methylethyl)-9-oxo-5,12-Epoxybenzocyclodecen-6-yl butanoate	-	-	-	-	-	tr	tr	++	+++	[48]
67.	Eunicellin diterpenoids	29.02	423.2742	363.2531<br>301.2167<br>285.2218<br>267.211<br>243.1746<br>229.1955<br>215.1798<br>201.1639<br>187.1484<br>163.1483<br>147.1171<br>141.0911<br>127.0754<br>107.0856<br>95.0856<br>81.0699	$C_{24}H_{38}O_6$<br>(-0.5)	Cladieunicellin K	-	tr	tr	tr	-	tr	-	+++	+++	[49]
68.	Sterols	29.15	413.3405	395.331<br>339.2691<br>309.2577<br>271.2059<br>253.1952<br>241.1952<br>227.1795<br>213.164<br>199.1483<br>185.1328<br>165.091<br>159.1168<br>141.1274<br>133.1012<br>123.117<br>109.1013<br>95.0857	$C_{28}H_{44}O_2$<br>(2.3)	3β-Hydroxyergosta-4,24(28)-diene-6-one	+	-	+	++	+++	-	-	-	-	[50]

				81.0699<br>69.0698											
69.	Eunicellin diterpenoids	29.32	389.2682	329.2088<br>301.2152<br>283.2059<br>199.1485<br>189.164<br>147.1172<br>137.133<br>119.0853<br>107.0855<br>93.0699	$C_{24}H_{36}O_4$<br>(0.7)	Litophynin G	tr	-	tr	tr	-	-	-	+++	+	[51]
70.	Eunicellin diterpenoids	29.62	449.2890	319.227<br>301.2163<br>283.2059<br>265.1955<br>255.211<br>241.1588<br>223.1482<br>215.1794<br>199.1482<br>185.1325<br>173.1324<br>157.1011<br>147.1167<br>131.0854<br>119.0855<br>107.0854<br>95.0491<br>81.0696<br>69.0697	$C_{26}H_{40}O_6$<br>(-4.0)	Litophynol A acetate isomer II	tr	tr	tr	tr	tr	tr	tr	-	+++	[25]
71.	Sterols	29.88	449.3616	431.352<br>413.3416<br>395.3311<br>377.3205<br>367.2993<br>287.2011<br>273.1853<br>251.2371<br>233.2268<br>175.1119<br>163.1483	$C_{28}H_{48}O_4$<br>(2.1)	Sarcrasterol	+	+	+	+++	+	+++	+	+	+	[52]

				149.1325<br>135.117<br>124.0427<br>109.1012<br>95.0856<br>81.0698<br>71.0855												
72.	Sterols	29.91	459.3462	441.3367<br>413.3415<br>395.3309<br>359.2586<br>217.1588<br>205.1223<br>177.0909<br>167.0701<br>145.1015<br>137.1322<br>123.117<br>109.1013<br>95.0855<br>81.0698<br>69.0697	$C_{29}H_{46}O_4$<br>(1.5)	Erectasteroid D	-	-	-	-	-	-	+	tr	tr	[53]
73.	Eunicellin diterpenoids	30.29	439.2684	379.248<br>337.2376<br>319.227<br>301.2164<br>291.2321<br>273.2217<br>263.1646<br>255.2112<br>221.1538<br>191.1432<br>175.1481<br>165.0911<br>137.1324<br>121.1012<br>109.1012<br>95.0855<br>81.0697	$C_{24}H_{38}O_7$<br>(1.5)	Cladieunicellin N	tr	tr	tr	-	tr	tr	-	+++	+	[54]
74.	Sterols	30.7	425.3407	385.235<br>201.1642<br>165.0911<br>145.1015<br>133.1012<br>123.1175<br>109.1011<br>95.0857<br>81.0697<br>69.0698	$C_{29}H_{44}O_2$<br>(1.7)	Stigmasta-5,8,22-trien-7-one, 3-hydroxy-,	-	-	-	-	-	-	+	-	tr	[55]
75.	Sterols	31.22	429.3359	393.316<br>369.2403<br>323.2372<br>309.2207<br>295.2065	$C_{28}H_{44}O_3$<br>(-1.8)	Dihydroxyergostadienon e	-	+	-	-	+++	-	+	-	-	[56]

				267.1742<br>227.1426<br>213.164<br>203.1791<br>189.1642<br>173.0962<br>165.091<br>147.0808<br>135.1167<br>121.0648<br>109.1011<br>97.1013<br>83.0857<br>69.0698												
76.	Eunicellin diterpenoids	31.76	453.2837	337.2375<br>319.2269<br>301.2164<br>255.2109<br>221.154<br>201.164<br>191.1433<br>183.1016<br>175.1485<br>165.0914<br>159.1169<br>153.0912<br>137.1326<br>121.1013<br>109.1012<br>95.0856<br>81.0699	$C_{25}H_{40}O_7$ (2.2)	Briarellin P	tr	tr	tr	tr	tr	-	tr	+++	tr	[57]
77.	Eunicellin diterpenoids	32.23	451.3049	391.2847<br>303.2322<br>285.2215<br>267.2109<br>225.164<br>215.1796<br>201.164<br>163.1482<br>147.1169<br>141.0911<br>127.0754<br>107.0855<br>95.0855<br>81.0698<br>71.049	$C_{26}H_{43}O_6$ (1.0)	Simplexin A	tr	-	tr	tr	-	tr	-	+++	+++	[58]
78.	Eunicellin diterpenoids	33.24	467.2999	319.2273<br>301.2167<br>191.1433<br>137.1326<br>121.1013<br>109.1013<br>95.0856<br>81.0698	$C_{26}H_{42}O_7$ (1.0)	Simplexin P	tr	tr	tr	tr	tr	tr	-	+++	+++	[59]

79.	Eunicellin diterpenoids	33.15	407.2782	345.2426<br>301.2163<br>283.206<br>269.2267<br>255.2114<br>229.1586<br>217.1588<br>199.1483<br>185.1325<br>171.1171<br>161.1326<br>149.0962<br>135.1169<br>121.1015<br>109.1014<br>93.0697<br>81.0699<br>69.0697	$C_{24}H_{38}O_5$ (2.4)	Australin G	tr	tr	tr	-	tr	-	tr	+++	tr	[60]

* Under the ESI positive conditions, certain eunicillin scaffolds may undergo the McLafferty rearrangement with a subsequent loss of H_2 forming the onium ion that in turn follows the typical fragmentation behavior of terpenes to finally offer valuable insights about the structure [61].

Table S2: Parameters used for the construction of the FBMN *via* the GNPS platform.

Precursor ion mass tolerance	0.5
Fragment ion mass tolerance	0.5
Cosine Score	0.7
Minimum matching fragments	6

References

1. Alam, M.; Sharma, P.; Zektzer, A. S.; Martin, G. E.; Ji, X.; van der Helm, D. Sclerophytin C-F: isolation and structures of four new diterpenes from the soft coral *Sclerophytum capitalis. J. Org. Chem.* **1989**, 54, 1896-1900.
2. Sugawara, T.; Zaima, N.; Yamamoto, A.; Sakai, S.; Noguchi, R.; Hirata, T. Isolation of sphingoid bases of sea cucumber cerebrosides and their cytotoxicity against human colon cancer cells. *Biosci. Biotechnol. Biochem.* **2006**, 0611080146-0611080146.
3. Wang, G.-H.; Ahmed, A. F.; Sheu, J.-H.; Duh, C.-Y.; Shen, Y.-C.; Wang, L.-T. Suberosols A− D, four new sesquiterpenes with β-caryophyllene skeletons from a Taiwanese gorgonian coral *Subergorgia suberosa*. *J. Nat. Prod.* **2002**, 65, 887-891.

4. Chen, D.; Chen, W.; Liu, D.; van Ofwegen, L.; Proksch, P.; Lin, W. Asteriscane-type sesquiterpenoids from the soft coral *Sinularia capillosa*. *J. Nat. Prod.* **2013**, 76, 1753-1763.
5. Elkhateeb, A.; El-Beih, A. A.; Gamal-Eldeen, A. M.; Alhammady, M. A.; Ohta, S.; Paré, P. W.; Hegazy, M.-E. F. New terpenes from the Egyptian soft coral *Sarcophyton ehrenbergi*. *Mar. Drugs* **2014**, 12, 1977-1986.
6. Li, G.; Zhang, Y.; Deng, Z.; van Ofwegen, L.; Proksch, P.; Lin, W. Cytotoxic Cembranoid Diterpenes from a Soft Coral *Sinularia gibberosa*. *J. Nat. Prod.* **2005**, 68, 649-652.
7. Anjaneyulu, A.; Chaturvedula, V. S. P. New Sesqui-and Diterpenoids from the Soft Coral *Nephthea chabroli* of Indian Coast. *Indian J. Chem. Sect. B* **2013**, 34B, 32.
8. Coll, J. C.; Bowden, B. F.; Tapiolas, D. M.; Willis, R. H.; Djura, P.; Streamer, M.; Trott, L. Studies of Australian soft corals—XXXV: The terpenoid chemistry of soft corals and its implications. *Tetrahedron* **1985**, 41, 1085-1092.
9. Chen, B.-W.; Chang, S.-M.; Huang, C.-Y.; Chao, C.-H.; Su, J.-H.; Wen, Z.-H.; Hsu, C.-H.; Dai, C.-F.; Wu, Y.-C.; Sheu, J.-H. Hirsutalins A− H, eunicellin-based diterpenoids from the soft coral *Cladiella hirsuta*. *J. Nat. Prod.* **2010**, 73, 1785-1791.
10. Cheng, S.-Y.; Chuang, C.-T.; Wen, Z.-H.; Wang, S.-K.; Chiou, S.-F.; Hsu, C.-H.; Dai, C.-F.; Duh, C.-Y. Bioactive norditerpenoids from the soft coral *Sinularia gyrosa*. *Bioorg. Med. Chem.* **2010**, 18, 3379-3386.
11. Cheng, Y.-B.; Shen, Y.-C.; Kuo, Y.-H.; Khalil, A. T. Cembrane diterpenoids from the Taiwanese soft coral *Sarcophyton stolidotum*. *J. Nat. Prod.* **2008**, 71, 1141-1145.
12. Sawant, S. S.; Youssef, D. T. A.; Reiland, J.; Ferniz, M.; Marchetti, D.; El Sayed, K. A. Biocatalytic and antimetastatic studies of the marine cembranoids sarcophine and 2-epi-16-deoxysarcophine. *J. Nat. Prod.* **2006**, 69, 1010-1013.
13. Zheng, J.-J.; Shao, C.-L.; Chen, M.; Gan, L.-S.; Fang, Y.-C.; Wang, X.-H.; Wang, C.-Y. Ochracenoids A and B, guaiazulene-based analogues from gorgonian *Anthogorgia ochracea* collected from the South China Sea. *Mar. Drugs* **2014**, 12, 1569-1579.
14. Lai, D.; Liu, D.; Deng, Z.; van Ofwegen, L.; Proksch, P.; Lin, W. Antifouling eunicellin-type diterpenoids from the gorgonian *Astrogorgia* sp. *J. Nat. Prod.* **2012**, 75, 1595-1602.
15. Hegazy, M.-E. F.; Elshamy, A. I.; Mohamed, T. A.; Hamed, A. R.; Ibrahim, M. A.; Ohta, S.; Paré, P. W. Cembrene diterpenoids with ether linkages from *Sarcophyton ehrenbergi*: An anti-proliferation and molecular-docking assessment. *Mar. Drugs* **2017**, 15, 192.
16. Cheng, S.-Y.; Wang, S.-K.; Wen, Z.-H.; Dai, C.-F.; Duh, C.-Y. Three new eudesmanoids from the Formosan soft coral *Nephthea erecta*. *J. Asian Nat. Prod. Res.* **2009**, 11, 967-973.
17. Cheng, S.-Y.; Huang, Y.-C.; Wen, Z.-H.; Chiou, S.-F.; Wang, S.-K.; Hsu, C.-H.; Dai, C.-F.; Duh, C.-Y. Novel sesquiterpenes and norergosterol from the soft corals *Nephthea erecta* and *Nephthea chabroli*. *Tetrahedron Lett.* **2009**, 50, 802-806.
18. Chang, Y.-C.; Huang, I.-C.; Chiang, M. Y.-N.; Hwang, T.-L.; Kung, T.-H.; Lin, C.-S.; Sheu, J.-H.; Sung, P.-J. Briaviodiol A, a new cembranoid from a soft coral *Briareum violacea*. *Chem. Pharm. Bull.* **2010**, 58, 1666-1668.
19. Chen, Y.-F.; Chen, W.-F.; Wen, Z.-H.; Hwang, T.-L.; Zhang, Z.-J.; Sung, P.-J. New bioactive $\Delta^{11(17)}$-furanoeunicellins from an octocoral *Cladiella* sp. *Phytochem. Lett.* **2019**, 33, 31-35.
20. Wanzola, M.; Furuta, T.; Kohno, Y.; Fukumitsu, S.; Yasukochi, S.; Watari, K.; Tanaka, C.; Higuchi, R.; Miyamoto, T. Four new cembrane diterpenes isolated from an Okinawan soft coral *Lobophytum crassum* with inhibitory effects on nitric oxide production. *Chem. Pharm. Bull.* **2010**, 58, 1203-1209.
21. Hassan, H. M.; Khanfar, M. A.; Elnagar, A. Y.; Mohammed, R.; Shaala, L. A.; Youssef, D. T.; Hifnawy, M. S.; El Sayed, K. A. Pachycladins A− E, prostate cancer invasion and migration inhibitory eunicellin-based diterpenoids from the Red Sea soft coral *Cladiella pachyclados*. *J. Nat. Prod.* **2010**, 73, 848-853.

22. Liang, L.-F.; Gao, L.-X.; Li, J.; Taglialatela-Scafati, O.; Guo, Y.-W. Cembrane diterpenoids from the soft coral *Sarcophyton trocheliophorum* Marenzeller as a new class of PTP1B inhibitors. *Bioorg. Med. Chem.* **2013**, 21, 5076-5080.
23. Cheng, S.-Y.; Chen, P.-W.; Chen, H.-P.; Wang, S.-K.; Duh, C.-Y. New cembranolides from the Dongsha atoll soft coral *Lobophytum durum*. *Mar. Drugs* **2011**, 9, 1307-1318.
24. Mohamed, T. A.; Elshamy, A. I.; Abdel-Tawab, A. M.; AbdelMohsen, M. M.; Ohta, S.; Pare, P. W.; Hegazy, M.-E. F. Oxygenated Cembrene Diterpenes from *Sarcophyton convolutum:* Cytotoxic Sarcoconvolutum A-E. *Mar. Drugs* **2021**, 19, 519.
25. Cai, Y.-S.; Yao, L.-G.; Di Pascale, A.; Irace, C.; Mollo, E.; Taglialatela-Scafati, O.; Guo, Y.-W. Polyoxygenated diterpenoids of the eunicellin-type from the Chinese soft coral *Cladiella krempfi*. *Tetrahedron* **2013**, 69, 2214-2219.
26. Yin, S.-W.; He, X.-M.; Wang, B.-G. Chemical Constituents of *Lobophytum crassum* and Their Cytotoxic Activity. *Acta Agriculturae Universitatis Jiangxiensis* **2008**.
27. Wang, C.-Y.; Chen, A.-N.; Shao, C.-L.; Li, L.; Xu, Y.; Qian, P.-Y. Chemical constituents of soft coral *Sarcophyton infundibuliforme* from the South China Sea. *Biochem. Syst. Ecol.* **2011**, 39, 853-856.
28. Rodríguez, A. D.; González, E.; González, C. Additional dolabellane diterpenes from the Caribbean gorgonian octocoral *Eunicea laciniata*. *J. Nat. Prod.* **1995**, 58, 226-232.
29. Zhang, C.-X.; He, X.-X.; Zhang, J.; Guo, Q.; Lei, L.-F.; Su, J.-Y.; Zeng, L.-M. New precursor of tetraterpenoids from the soft coral *Sarcophyton glaucum*. *Nat. Prod. Res.* **2013,** 27, 782-786.
30. Lin, M.-C.; Chen, B.-W.; Huang, C.-Y.; Dai, C.-F.; Hwang, T.-L.; Sheu, J.-H. Eunicellin-based diterpenoids from the Formosan soft coral *Klyxum molle* with inhibitory activity on superoxide generation and elastase release by neutrophils. *J. Nat. Prod.* **2013**, 76, 1661-1667.
31. Li, G.; Li, H.; Zhang, Q.; Yang, M.; Gu, Y.-C.; Liang, L.-F.; Tang, W.; Guo, Y.-W. Rare cembranoids from Chinese soft coral *Sarcophyton ehrenbergi*: Structural and stereochemical studies. *J. Org. Chem.* **2019**, 84, 5091-5098.
32. Abou El-Ezz, R. F.; Ahmed, S. A.; Radwan, M. M.; Ayoub, N. A.; Afifi, M. S.; Ross, S. A.; Szymanski, P. T.; Fahmy, H.; Khalifa, S. I. Bioactive cembranoids from the Red Sea soft coral *Sarcophyton glaucum*. *Tetrahedron Lett.* **2013**, 54, 989-992.
33. Chen, S.-P.; Chen, B.-W.; Dai, C.-F.; Sung, P.-J.; Wu, Y.-C.; Sheu, J.-H. Sarcophytonins F and G, new dihydrofuranocembranoids from a Dongsha Atoll soft coral *Sarcophyton* sp. *Bull. Chem. Soc. Jpn.* **2012**, 85, 920-922.
34. Rao, C. B.; Rao, D. S.; Satyanarayana, C.; Rao, D. V.; Kassühlke, K. E.; Faulkner, D. J. New cladiellane diterpenes from the soft coral *Cladiella australis* of the Andaman and Nicobar Islands. *J. Nat. Prod.* **1994**, 57, 574-580.
35. Ahmed, S.; Ibrahim, A.; Arafa, A. S. Anti-H5N1 virus metabolites from the Red Sea soft coral, *Sinularia candidula*. *Tetrahedron Lett.* **2013**, 54, 2377-2381.
36. Lai, D.; Li, Y.; Xu, M.; Deng, Z.; Van Ofwegen, L.; Qian, P.; Proksch, P.; Lin, W. Sinulariols A–S, 19-oxygenated cembranoids from the Chinese soft coral *Sinularia rigida. Tetrahedron* **2011**, 67, 6018-6029.
37. Duh, C.-Y.; Wang, S.-K.; Chu, M.-J.; Sheu, J.-H. Cytotoxic sterols from the soft coral *Nephthea erecta*. *J. Nat. Prod.* **1998**, 61, 1022-1024.
38. Chen, B.-W.; Wu, Y.-C.; Chiang, M. Y.; Su, J.-H.; Wang, W.-H.; Fan, T.-Y.; Sheu, J.-H. Eunicellin-based diterpenoids from the cultured soft coral *Klyxum simplex*. *Tetrahedron* **2009**, 65, 7016-7022.
39. Lin, W.-Y.; Su, J.-H.; Lu, Y.; Wen, Z.-H.; Dai, C.-F.; Kuo, Y.-H.; Sheu, J.-H. Cytotoxic and anti-inflammatory cembranoids from the Dongsha Atoll soft coral *Sarcophyton crassocaule*. *Bioorg. Med. Chem.* **2010**, 18, 1936-1941.

40. Maloney, K. N.; Botts, R. T.; Davis, T. S.; Okada, B. K.; Maloney, E. M.; Leber, C. A.; Alvarado, O.; Brayton, C.; Caraballo-Rodríguez, A. M.; Chari, J. V. Cryptic species account for the seemingly idiosyncratic secondary metabolism of *Sarcophyton glaucum* specimens collected in Palau. *J. Nat. Prod.* **2020**, 83, 693-705.
41. Hegazy, M.-E. F.; Eldeen, A. M. G.; Shahat, A. A.; Abdel-Latif, F. F.; Mohamed, T. A.; Whittlesey, B. R.; Paré, P. W. Bioactive hydroperoxyl cembranoids from the Red Sea soft coral *Sarcophyton glaucum*. *Mar. Drugs* **2012**, 10, 209-222.
42. Kobayashi, M.; Iesaka, T.; Nakano, E. Marine Terpenes and Terpenoids. IX.: Structures of Six New Cembranoids, Sarcophytols F, K, P, Q, R and S, from the Soft Coral *Sarcophyton glaucum*. *Chem. Pharm. Bull.* **1989**, 37, 2053-2057.
43. Li, J. F.; Zeng, Y. B.; Li, W. S.; Luo, H.; Zhang, H. Y.; Guo, Y. W. Xishaglaucumins A—J, New Cembranoids with Anti-Inflammatory Activities from the South China Sea Soft Coral *Sarcophyton glaucum*. *Chin. J. Chem.* **2022**, 40, 79-90.
44. Zhao, M.; Li, X.; Zhao, F.; Cheng, S.; Xiang, Z.; Dong, J.; Huang, K.; Yan, P. Four new 7, 8-epoxycembranoids from a Chinese soft coral *Lobophytum* sp. *Chem. Pharm. Bull.* **2013**, 61, 1323-1328.
45. Ahmed, A. F.; Chen, Y.-W.; Huang, C.-Y.; Tseng, Y.-J.; Lin, C.-C.; Dai, C.-F.; Wu, Y.-C.; Sheu, J.-H. Isolation and structure elucidation of cembranoids from a Dongsha Atoll soft coral *Sarcophyton stellatum*. *Mar. Drugs* **2018**, 16, 210.
46. Zhang, Z.-J.; Wang, Y.-H.; Chen, S.-R.; Peng, B.-R.; Yang, S.-N.; Hu, C.-C.; Fang, L.-S.; Hwang, T.-L.; Sung, P.-J. Novel secoeunicellins produced by an octocoral *Cladiella* sp. *Tetrahedron Lett.* **2019**, 60, 151300.
47. Li, Y.; Gao, A. H.; Huang, H.; Li, J.; Mollo, E.; Gavagnin, M.; Cimino, G.; Gu, Y. C.; Guo, Y. W. Diterpenoids from the Hainan soft coral *Sinularia parva*. *Helv. Chim. Acta* **2009**, 92, 1341-1348.
48. Iwagawa, T.; Kusatsu, T.; Tsuha, K. Hamada, T.; Okamura, H.; Furukawa, T.; Akiyama, S.-I.; Doe, M.; Morimoto, Y.; Iwase, F.; Takemura, K. Cytotoxic eunicellin-type diterpenes from the soft coral *Litophyton viscudium*. *Heterocycles* **2011**, 83, 2149-2155.
49. Shih, F.-Y.; Chen, T.-H.; Lu, M.-C.; Chen, W.-F.; Wen, Z.-H.; Kuo, Y.-H.; Sung, P.-J. Cladieunicellins K and L, new eunicellin-based diterpenoids from an octocoral *Cladiella* sp. *Int. J. Mol. Sci.* **2013**, 14, 21781-21789.
50. Ngoc, N. T.; Huong, P. T. M.; Van Thanh, N.; Chi, N. T. P.; Dang, N. H.; Cuong, N. X.; Nam, N. H.; Thung, D. C.; van Kiem, P.; van Minh, C. Cytotoxic steroids from the Vietnamese soft coral *Sinularia conferta*. *Chem. Pharm. Bull.* **2017**, 65, 300-305.
51. Ochi, M.; Yamada, K.; Futatsugi, K.; Kotsuki, H.; Shibata, K. Litophynins F, G, and H, Three New Diterpenoids from a Soft Coral *Litophyton* sp. *Heterocycles* **1991**, 32, 29-32.
52. Zhang, C.-X.; Yan, S.-J.; Zhang, G.-W.; Su, J.-Y.; Zeng, L.-M. Isolation of new polyhydroxylated sterol from soft coral *Sarcophyton crassocaule* Mosre. *Chem. J. Chin. Univ.* **2007**, 28, 686-688.
53. Cheng, S.-Y.; Dai, C.-F.; Duh, C.-Y. New 4-methylated and 19-oxygenated steroids from the Formosan soft coral *Nephthea erecta. Steroids* **2007**, 72, 653-659.
54. Chen, T.-H.; Chen, W.-F.; Wen, Z.-H.; Lu, M.-C.; Wang, W.-H.; Li, J.-J.; Wu, Y.-C.; Sung, P.-J. Cladieunicellins M–Q, new eunicellins from *Cladiella* sp. *Mar. Drugs* **2014**, 12, 2144-2155.
55. Songzhi, D.; Chunlei, T.; Dingjun, X. Studies on the chemical constituents of the sponge *Biemna fortis* from the South China Sea. *Chin. J. Mar. Drugs* **1999**, 18, 4-6.
56. Zhang, W.; Liu, W. K.; Che, C.-T. Polyhydroxylated steroids and other constituents of the soft coral *Nephthea chabroli*. *Chem. Pharm. Bull.* **2003**, 51, 1009-1011.
57. Ospina, C. A.; Rodríguez, A. D.; Ortega-Barria, E.; Capson, T. L. Briarellins J– P and Polyanthellin A: New Eunicellin-Based Diterpenes from the Gorgonian Coral *Briareum polyanthes* and Their Antimalarial Activity. *J. Nat. Prod.* **2003**, 66, 357-363.

58. Wu, S.-L.; Su, J.-H.; Wen, Z.-H.; Hsu, C.-H.; Chen, B.-W.; Dai, C.-F.; Kuo, Y.-H.; Sheu, J.-H. Simplexins A− I, eunicellin-based diterpenoids from the soft coral *Klyxum simplex*. *J. Nat. Prod.* **2009**, 72, 994-1000.
59. Wu, S.-L.; Su, J.-H.; Huang, C.-Y.; Tai, C.-J.; Sung, P.-J.; Liaw, C.-C.; Sheu, J.-H. Simplexins P–S, eunicellin-based diterpenes from the soft coral *Klyxum simplex*. *Mar. Drugs* **2012**, 10, 1203-1211.
60. Williams, D. E.; Amlani, A.; Dewi, A. S.; Patrick, B. O.; van Ofwegen, L.; Mui, A. L.-F.; Andersen, R. J. Australin E Isolated from the Soft Coral *Cladiella* sp. Collected in Pohnpei Activates the Inositol 5-Phosphatase SHIP1. *Aust. J. Chem.* **2010**, 63, 895-900.
61. Kyeremeh, K.; Baddeley, T. C.; Stein, B. K.; Jaspars, M. A homologous series of eunicellin-based diterpenes from *Acalycigorgia* sp. characterised by tandem mass spectrometry. *Tetrahedron* **2006**, 62, 8770-8778.
